# Genetic diversity and selective sweeps in historical and modern Canadian spring wheat cultivars using the 90K SNP array

**DOI:** 10.1038/s41598-021-02666-5

**Published:** 2021-12-10

**Authors:** Kassa Semagn, Muhammad Iqbal, Nikolaos Alachiotis, Amidou N’Diaye, Curtis Pozniak, Dean Spaner

**Affiliations:** 1 Department of Agricultural, Food, and Nutritional Science, 4-10 Agriculture-Forestry Centre, University of Alberta, Edmonton, AB T6G 2P5 Canada; 2 Faculty of Electrical Engineering, Mathematics and Computer Science, University of Twente, 3230 Enschede, OV The Netherlands; 3 Crop Development Centre and Department of Plant Sciences, University of Saskatchewan, 51 Campus Drive, Saskatoon, SK S7N 5A8 Canada

**Keywords:** Genetics, Agricultural genetics

## Abstract

Previous molecular characterization studies conducted in Canadian wheat cultivars shed some light on the impact of plant breeding on genetic diversity, but the number of varieties and markers used was small. Here, we used 28,798 markers of the wheat 90K single nucleotide polymorphisms to (a) assess the extent of genetic diversity, relationship, population structure, and divergence among 174 historical and modern Canadian spring wheat varieties registered from 1905 to 2018 and 22 unregistered lines (hereinafter referred to as cultivars), and (b) identify genomic regions that had undergone selection. About 91% of the pairs of cultivars differed by 20–40% of the scored alleles, but only 7% of the pairs had kinship coefficients of < 0.250, suggesting the presence of a high proportion of redundancy in allelic composition. Although the 196 cultivars represented eight wheat classes, our results from phylogenetic, principal component, and the model-based population structure analyses revealed three groups, with no clear structure among most wheat classes, breeding programs, and breeding periods. F_ST_ statistics computed among different categorical variables showed little genetic differentiation (< 0.05) among breeding periods and breeding programs, but a diverse level of genetic differentiation among wheat classes and predicted groups. Diversity indices were the highest and lowest among cultivars registered from 1970 to 1980 and from 2011 to 2018, respectively. Using two outlier detection methods, we identified from 524 to 2314 SNPs and 41 selective sweeps of which some are close to genes with known phenotype, including plant height, photoperiodism, vernalization, gluten strength, and disease resistance.

## Introduction

Canada is one of the top ten wheat producers and exporters globally with nearly 92% of its wheat produced in the three prairies provinces of Alberta, Saskatchewan, and Manitoba (https://www150.statcan.gc.ca/). Hexaploid spring wheat (*Triticum aestivum* L.) is the dominant type of wheat accounting for ~ 72% of the total wheat production in 2020 in the country, followed by 23% of durum wheat [*Triticum turgidum* L. ssp. *durum* (Desf.) Husn.], and 5% of winter wheat (https://www150.statcan.gc.ca/). A total of 363 spring wheat varieties (cultivars) have been registered in Canada by 34 representative institutions and companies (https://inspection.canada.ca/active/netapp/regvar/regvar_lookupe.aspx) of which 260 spring wheat varieties had regional registration, including 172 registered for production in the British Columbia and the three prairies provinces. Currently, spring wheat varieties in Western Canada have been classified into eight wheat (market) classes based on grain characteristics (e.g., color, hardness, size), baking quality (dough/gluten strength), milling quality, grain protein concentration, and their end-use (https://www.grainscanada.gc.ca/en/grain-quality/grain-grading/wheat-classes.html). The eight classes are Canada Northern Hard Red (CNHR), Canada Prairie Spring Red (CPSR), Canada Prairie Spring White (CPSW), Canada Western Extra Strong (CWES), Canada Western Hard White Spring (CWHWS), Canada Western Red Spring (CWRS), Canada Western Soft White Spring (CWSWS), and Canada Western Special Purpose (CWSP). Several varieties have been reclassified recently, which could have a significant financial impact on seed growers who have been multiplying pedigreed stocks to sell them as certified seed. We were interested to assess the extent of genetic differentiation (divergence) among these wheat classes, which forms one of the bases of this study.

The impact of plant breeding on the genetic diversity of crops has been studied using diverse types of molecular markers, including simple sequence repeats (SSRs) or microsatellites^1–4^, expressed sequence tags^5,6^, and single nucleotide polymorphisms (SNP) generated through different genotyping platforms^7–10^. Results of the various molecular diversity studies shed some light on the impact of plant breeding on crops’ genetic diversity, but the results are inconsistent with the general perception that modern plant breeding reduces crop genetic diversity. Some studies reported a wider genetic base and an increase in genetic diversity^2,3,9,11^, while others reported a narrow genetic base and reduced genetic diversity in most modern varieties^1,12,13^. For example, researchers at Wageningen University studied the genetic diversity of 90 tomato varieties introduced in the Netherlands from 1950 to 2016 using 7661 polymorphic SNPs. The authors found out that the current genetic diversity among the tomato varieties was eight times higher than those varieties grown from 1950 to 1960. The main reason cited by the authors was the introgression of many new disease resistance genes from wild relatives in modern varieties, which has occurred across the whole tomato genome^9^. Using meta-analyses of 44 published papers on genetic diversity of eight crops (of which 25 papers were in wheat), researchers at the Wageningen University have also demonstrated the lack of substantial reduction in genetic diversity of crop varieties released since the 1970s^11^. However, the authors reported a significant reduction in genetic diversity between 1950 and 1960 as have also been reported in 75 Nordic spring wheat cultivars adopted by farmers from 1901 to 1993^2^ and 511 winter wheat varieties that were widely grown in Central and Northern Europe between the 1940s and 1990s^3^. The study in the Nordic spring wheat cultivars demonstrated an increase in genetic diversity from 1901 to 1940 and again from 1960 onwards, but a loss of diversity in between the two periods.

In Canada, Agriculture and Agri-Food Canada (AAFC) researchers conducted multiple studies to assess the changes in the genetic diversity of 75 Canadian hard red wheat cultivars released from 1845 to 2004 using 31 SSR and 37 expressed sequence tags^4,5,12^ and then 370 SSRs^1,14^. The authors found a significant reduction in allelic diversity in every part of the wheat genome associated with long-term wheat improvement, but the extent of reduction in diversity differed based on the breeding periods and breeding programs. They also reported that selection for some traits, such as early maturity introduced more new alleles, but other traits resulted in the loss of alleles^1^. However, the number of varieties and markers used in their studies was small, which forms another basis in this study. Positive selection increases the frequency of beneficial alleles to reach fixation in the population, which leads to reduction of genetic variation among nucleotide sequences that are near the selected genome known as selective sweeps^15–17^. Such events increase the fitness of the individuals carrying it but reduce overall genetic diversity at the specific regions that have undergone selection^18–20^. Detecting traces of positive selection in the genome has been achieved by searching for loci that show reduced genetic variation, a shift in the site frequency spectrum, and particular linkage disequilibrium (LD) patterns in a particular region^21^. Different studies used a wide range of statistical methods of varying complexity to identify loci and genomic regions that have undergone selection, including the F-statistics based hierarchical island model in Arlequin^22^, the total variance F_ST_-based outlier detection method in Lositan^23^, the Bayesian genome scan method in BayeScan^24^, RAiSD^15^, SweeD^25^, SweepFinder^26^, SweepFinder2^27^, and OmegaPlus^28^. The first three methods (Arlequin, Lositan, and BayeScan) identify loci that have undergone selection by computing summary statistics on individual markers as compared with the other five methods that utilize advanced algorithms to scan the whole genome using physical information. The objectives of this study were, therefore, to (i) assess the molecular diversity, population structure, and genetic relationship among historical and modern Canadian spring wheat cultivars; (ii) compare the extent of molecular diversity indices and genetic differentiation (divergence) among different categorical variables (predicted groups, wheat classes, breeding periods, and breeding programs), and (iii) detect SNPs and genomic regions that have undergone selection using two contrasting outlier detection methods and explore if some of those regions are physically close to known genes that regulate phenotypic traits.

## Methods

We used a total of 196 genotypes consisting of 174 spring wheat varieties (cultivars) registered in western Canada between 1905 and 2018 (174) and 22 unregistered lines (Supplementary Table S1, Supplementary Fig. S1). Because nearly 89% of the samples used in the present study were cultivars and 11% lines, all genotypes hereinafter are referred to as cultivars. The methodologies for DNA extraction, normalization, and genotyping have been described in our previous studies^29,30^. Briefly, the cultivars were genotyped at the University of Saskatchewan wheat genomics lab, Saskatoon, Canada, with the wheat 90K iSelect array, which consisted of a total of 81,587 SNPs^31^. We filtered the genotype data using a minor allele frequency (MAF) of 0.01, maximum heterozygosity of 50%, and missing data points of 20%, which resulted in 28,798 polymorphic SNPs for statistical analyses (Table 1, Supplementary Table S2). For each SNP, we obtained the International Wheat Genome Sequencing Consortium (IWGSC) RefSeq v2.0 physical map in two steps as described in our previous study^32^. First, we used the marker name to retrieve at least 100 bp of the original sequence at http://download.txgen.tamu.edu/shichen/flanking_v2.html. Next, we used the sequences for BLAST searches against IWGSC RefSeq v2.0, which is available at http://download.txgen.tamu.edu/shichen/mapper_v2.html and http://wheat-urgi.versailles.inra.fr/. The top hits with the highest alignment length and highest similarity (> 95%) were retrieved as described elsewhere^33^ and the start position was used to represent the physical position of each marker. Some of the SNPs did not either return hit or returned ambiguous hits that didn’t meet the blast search criteria; these SNPs were considered as unmapped.

**Table 1:** Summary of the chromosomal distribution and physical map length of 28,798 polymorphic SNPs used in the present study.

Genome	Chrom	No. of SNPs	Physical map length (bp)
A genome	1A	2082	597,830,412
A genome	2A	2219	787,699,648
A genome	3A	1564	753,387,692
A genome	4A	1397	754,061,530
A genome	5A	1701	712,381,302
A genome	6A	1626	622,543,160
A genome	7A	1884	744,464,802
B genome	1B	1993	700,533,516
B genome	2B	2319	812,709,623
B genome	3B	1570	851,869,969
B genome	4B	913	673,444,876
B genome	5B	1867	714,561,761
B genome	6B	1373	730,887,061
B genome	7B	1265	763,472,158
D genome	1D	846	498,396,697
D genome	2D	896	656,389,786
D genome	3D	521	619,453,042
D genome	4D	323	518,210,289
D genome	5D	598	569,783,883
D genome	6D	535	495,188,882
D genome	7D	548	642,776,311
Unmapped	Unmapped	758	
Total		28,798	14,220,046,400

See Supplementary Table S2 for details.

Most statistical analyses were performed as described in previous studies^34–36^. Population structure was examined using the Bayesian model-based methods implemented in STRUCTURE v2.3.4^37^. STRUCTURE was run by varying the number of clusters (K) between two and eight using an admixture model, a burn-in, and a Markov Chain Monte Carlo cycle of 50,000 for three replications. We used STRUCTURE HARVESTER^38^ for identifying the optimal number of clusters (groups) based on the Δ*K* method^39^. Cultivars with a probability of membership of ≥ 60% were assigned to the same group, while those with < 60% probability in any cluster were assigned to a ‘‘mixed’’ group^36^. We used Structure Plot^40^ v2 to render STRUCTURE bar plots. We assessed the extent of genetic differentiation among the different categorical variables (see below) using an analysis of molecular variance^41^ implemented in Arlequin version 3.5.2.2^22^. We also computed fixation index (F_ST_)-based pairwise genetic distance matrices^42^ across the different categorical variables using Arlequin. F_ST_ values are indicative of the extent of genetic divergence (differentiation) among groups, with < 0.05, 0.05–0.15, 0.15–0.25, and > 0.25 indicating little, moderate, great, and very great genetic differentiation^43^. We also computed different diversity indices among different categorical variables using the Tajima’s neutrality test^44^ implemented in Molecular Evolutionary Genetics Analysis (MEGA) X^45^, which included the number of polymorphic (segregating) sites (S), the proportion of polymorphic sites (Ps), Theta (θ), and nucleotide diversity (π) as described in the previous studies^34,35,46^.

We used multiple categorical variables for comparisons, including predicted group membership based on the model-based STRUCTURE, wheat classes effective as of 1st August 2021, representative breeding programs (institutions) that developed or registered the cultivars, and the breeding periods (year of registration or development) of the cultivars. We first divided the breeding periods into six corresponding to the pre-1970s, 1971–1980, 1981–1990, 1991–2000, 2001–2010, and 2011–2018. We also tried to assess the extent of genetic variation and divergence across four breeding periods by taking into account some of the rationale used in previous similar studies in Canada^4^ and the US^10^. The first period represents the cultivation of primarily introduced and old cultivars before the 1980s, followed by the second period that consisted of cultivars developed by breeders in Canada using conventional breeding methods between 1981 and 2000. The third period (2001–2010) corresponds to the first era of molecular breeding using low-density, low throughput, and laborious molecular markers, while the fourth period (2011–2018) corresponds to the availability of genotyping by sequencing technologies, which generates relatively low cost, high throughput, and high-density molecular markers for germplasm characterization and molecular breeding. One hundred seventy-four of the 196 cultivars were also assigned into six breeding programs based on the institution (companies) that registered/developed them, which included Agriculture and Agri-Food Canada (74 cultivars), University of Saskatchewan (29), University of Alberta (19), Secan Association (30), Syngenta Canada Inc. (14), and Nutrien AG Solutions Inc. (8). The remaining 22 cultivars came from ten breeding programs (institutions) and were assigned to “others” due to their small sample size. We computed relative kinship using TASSEL v4.2.0 and both identity by state (IBS)-based genetic distance and principal component analysis using TASSEL v5.2.72^47^. We constructed phylogenetic trees from the IBS-based distance matrices using the neighbor-joining method implemented in MEGA X^45^. The first three principal components from PCA were plotted for visual examination of the population structure in CurlyWhirly v1.19.09.04 (The James Hutton Institute, Information & Computational Sciences) using the different categorical variables.

To detect loci that may have undergone selection, we first examined the joint distribution of F_ST_ and heterozygosity under a hierarchical island model implemented in Arlequin^22^ using the different categorical variables. Markers were declared under selection by examining the distribution of F_ST_ values that are significantly (*p* < 0.05) different among categorical variables and greater than a threshold (target mean F_ST_). Second, we used RAiSD^15^ to detect selective sweeps that may have undergone selection without a priori knowledge as described in a previous study^15^. We used a chromosome-wise threshold score to select the top outliers as candidate selective sweeps, which account for about 0.1% of the initial number of markers used for analysis per chromosome. The threshold values for declaring selective sweeps varied from 6 on chromosome 1D and 4D to 35 on 3B. The start and end physical positions of each candidate selective sweep region were used to search for candidate genes at Ensembl Plants using *Triticum aestivum* database (https://plants.ensembl.org/index.html). The gene ID retrieved from Ensembl Plants was then used to search for predicted gene functions at the Triticeae Toolbox (https://triticeaetoolbox.org/wheat/). We computed the squared regression coefficient (r^2^) as a measure of LD between pairs of genome-wide SNPs and outlier SNPs identified by the hierarchical island model using a sliding window size of 35 markers in TASSEL v5.2.74. Locally Weighted Scatterplot Smoothing (LOESS) curves were fitted by plotting r^2^ values on the Y-axis against physical distance in mega base pairs on the X-axis using JMP v16 (https://www.jmp.com/). Chromosome-wise r^2^ values were visualized using both TASSEL.

## Results

### Marker polymorphism and population structure

Of the 81,587 SNPs used for genotyping the association mapping panel, only 35.3% were used in the final analysis, each with a minor allele frequency of ≥ 0.01; other SNPs were either monomorphic or had > 20% missing data. The number of polymorphic SNPs per chromosome varied from 323 on chromosome 4D to 2319 on 2B (Table 1), with an overall average of 1335 SNPs. Nearly 43%, 39%, and 15% of the polymorphic SNPs belonged to the A, B, and D genomes, respectively. The physical length of each chromosome varied from 495.2 Mb on chromosome 6D to 851.9 Mb on 3B. Seven hundred fifty-eight of the polymorphic SNPs that account for 2.6% of the markers were not physically mapped. The proportion of missing data per polymorphic marker varied from 0 to 19.8%, with an overall average of 5% missing. Minor allele frequency per marker varied from 0.01 to 0.50 and the overall average was 0.21. About 47% of the SNPs had a minor allele frequency greater than the overall average (Supplementary Table S2).

**Figure 1:**
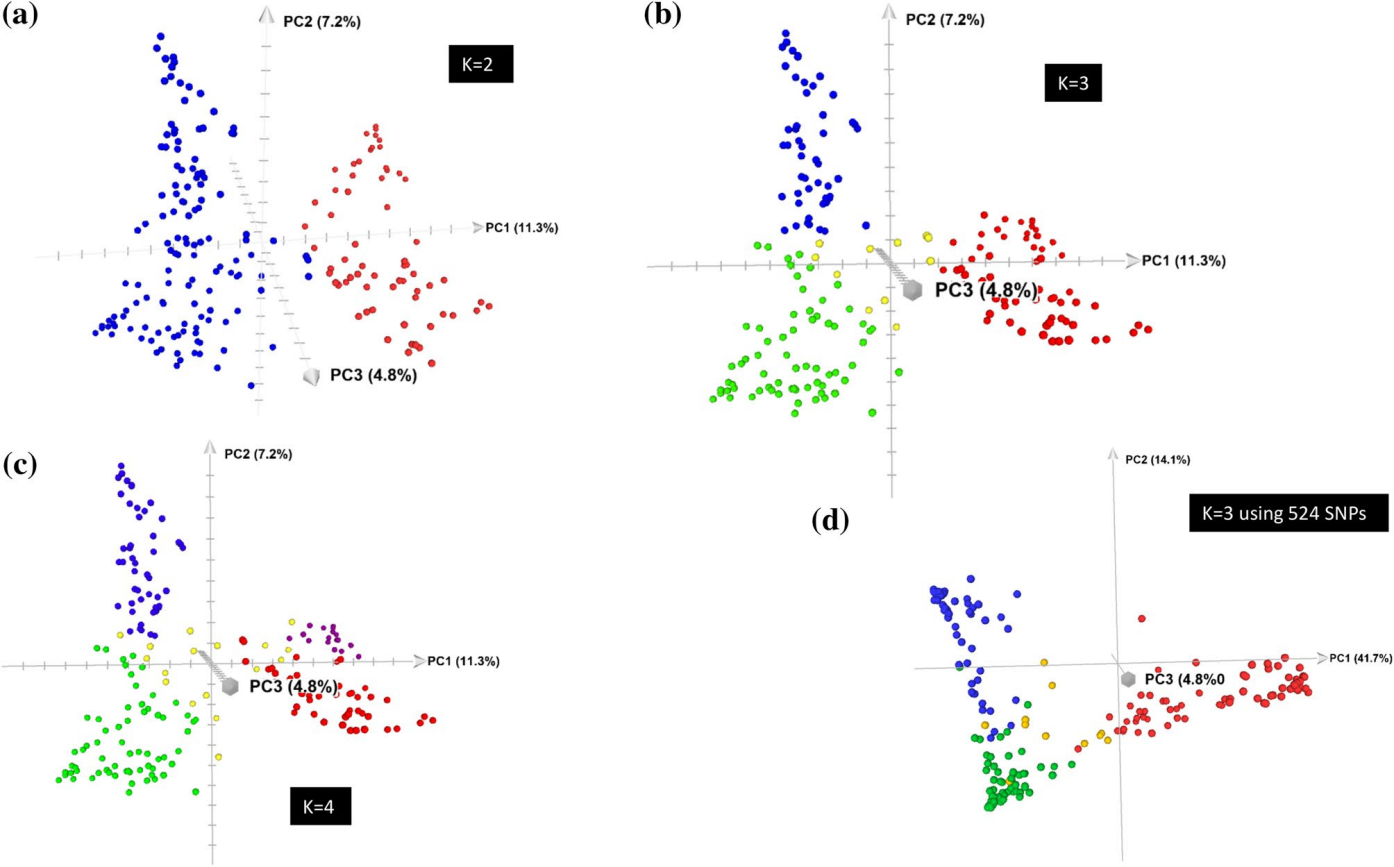
(**a**–**c**) Plots of PC1 (11.3% of variation), PC2 (7.2%), and PC3 (4.8%) from principal component analyses of 196 spring wheat genotyped with 28,798 polymorphic SNPs. The plots are based on predicted group membership from STRUCTURE at K = 2, K = 3, and K = 4. (**d**) A plot of PC1 (41.7% of variation), PC2 (14.1%), and PC3 (4.8%) from principal component analyses of 196 spring wheat genotyped with 524 of the 28,798 polymorphic SNPs that have undergone selection across the three predicted groups. Genotypes belong to the same group are shown with the same font—Group-1 (blue), group-2 (red), group-3 (green), group-4 (purple), and mixed (yellow).

**Figure 2:**
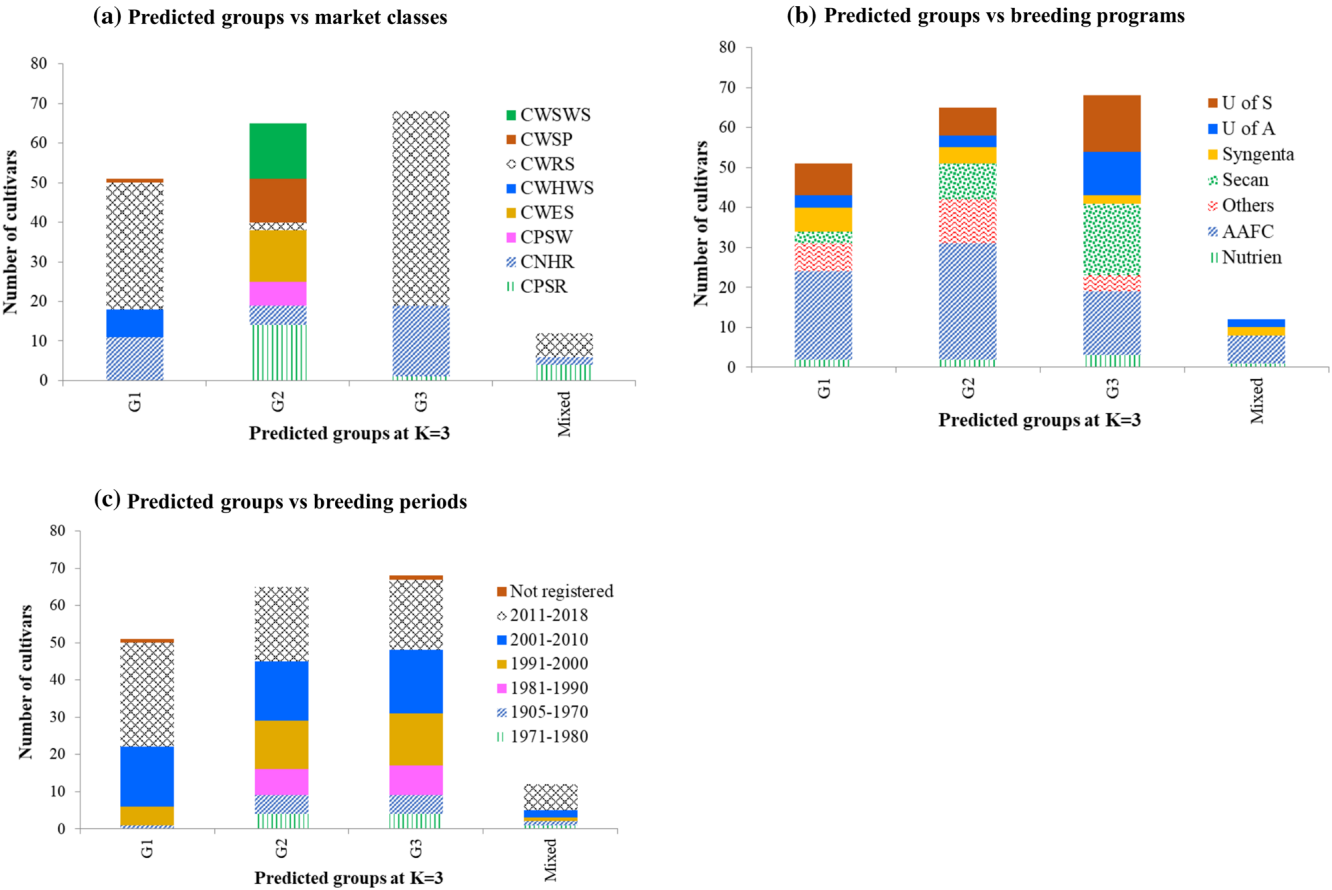
Summary of population structure of 196 spring wheat cultivars based on 28,798 polymorphic SNPs at K = 3: (**a**) group membership based on the eight wheat classes; (**b**) group membership based on six breeding programs (representative institutions/companies); and (**c**) group membership based on six breeding periods.

**Figure 3:**
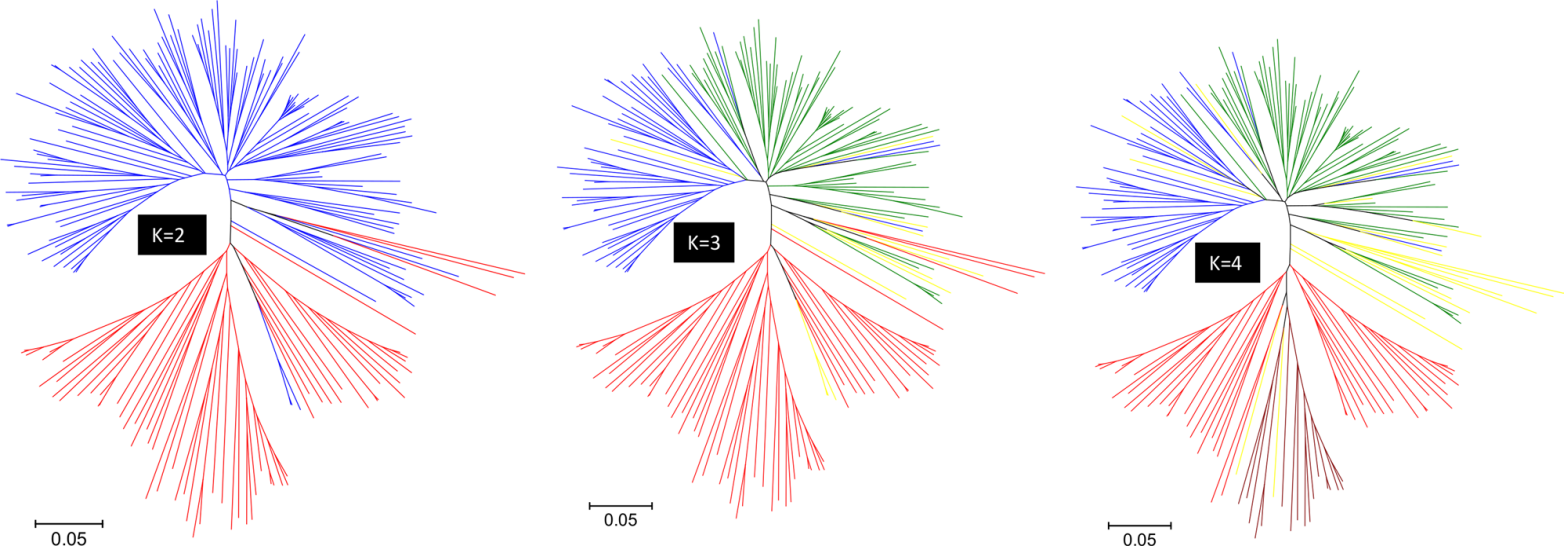
Neighbor-joining tree of 196 spring wheat genotyped with 28,798 polymorphic SNPs. The plots are based on predicted group membership from STRUCTURE at K = 2, K = 3, and K = 4. Genotypes belonging to the same group are shown with the same font—Group-1 (blue), group-2 (red), group-3 (green), group-4 (purple), and mixed (yellow).

We used the model-based population structure and PCA to determine how cultivars tended to cluster into groups. The LnP(D) sharply increased between K = 1 and K = 4, and mostly reached a plateau between K = 5 and K = 8, while the ad hoc statistic ∆K declined between K = 2 and K = 4, suggesting up to four possible groups (Supplementary Table S1 and Fig. S2). The first five principal components from PCA accounted for 30.3% of the genetic variation. To decide the most likely number of groups, we constructed a scatter plot of the first three principal components using the predicted group memberships from STRUCTURE, which is summarized in Fig. 1. The PCA plots showed three groups corresponding to the group membership predicted at K = 3. The first group consisted of a total of 51 cultivars from five wheat classes, which included all CWHWS (7 cultivars), CWRS (32), CNHR (11), and CWSP (1). The second group consisted of 65 cultivars belonging to seven wheat classes—CNHR (5), CPSR (14), CPSW (6), CWES (13), CWRS (2), CWSP (11), and CWSWS (14). The third group had 68 cultivars from the CWRS (49), CNHR (18), and CPSR (1) classes. Twelve cultivars from CNHR (2), CPSR (4), and CWRS (6) with < 60% probability in any group were assigned to a ‘‘mixed’’ group. Nearly 84% of the 51 cultivars in Group 1, 11% of the 65 cultivars in Group 2, and 99% of the 68 cultivars in Group 3 were CNHR and CWRS. CNHR consists of mainly cultivars moved from CWRS due to their low gluten strength plus a few CPSR cultivars. This classification is still ongoing and five of the CWRS cultivars (5605HR CL, AAC Redwater, AC Domain, Muchmore, and Vesper) and one of the CPSR cultivars (CDC Cordon CLPlus) were the recent addition into CNHR that came to effect as of 1st of August 2021. We did not find clear clustering patterns among breeding programs, and cultivar registration/development periods (Fig. 2, Supplementary Fig. S3). Phylogenetic trees constructed using the IBS-based genetic distance (Supplementary Table S3) and the different predicted group's membership from the model-based population structure analyses revealed three distinct groups (Fig. 3) similar to the predicted groups at K = 3, but there were no distinct patterns of clustering across most wheat classes, breeding programs, and breeding periods (Supplementary Fig. S4).

Table 2 summarizes the partitioning of the molecular variances among seven categorical variables: predicted groups based on STRUCTURE at K = 2, K = 3, and K = 4, the eight wheat classes, the six breeding programs, and four and six breeding periods. Differences among breeding periods and breeding programs accounted for just 2.5–3.1% of the genetic variation, which was very small as compared with the variance components attributed due to predicted groups (19.6–24.7%), and wheat classes (20.6%). Most of the molecular variance (75.3–97.6%) was observed within groups (population), which is expected in primarily self-pollinating species. F_ST_ values computed among categorical variables revealed little genetic differentiation (F_ST_ ≤ 0.050) among breeding programs and breeding periods (Supplementary Table S4). On the other hand, we found highly variable F_ST_ values among the three predicted groups (0.195–0.418) and the eight wheat classes (0.022–0.451). Of the 28 pairwise comparisons of F_ST_ values among the eight wheat classes, F_ST_ was the smallest (0.022) between CNHR and CWRS and the largest (0.451) between CPSW and CWHWS. The extent of genetic differentiation (divergence) among predicted groups and wheat classes is also very evident in the PCA plots (Fig. 1 and Supplementary Fig. S3) and phylogenetic tree (Fig. 3 and Supplementary Fig. S4).

**Table 2:** Analysis of molecular variance (AMOVA) for the extraction of SNP variation among and within groups (populations) based on 28,798 polymorphic SNPs.

Categorical variable	Source of variation	Degree of freedom	Sum of squares	Variance components	Percentage variation	F_ST_
**All 28,798 SNPs**						
STRUCTURE groups at K = 2	Between predicted groups	1	76,815.8	844.1	19.6	0.196
	Within groups	194	673,966.1	3474.1	80.5	
STRUCTURE groups at K = 3	Among predicted groups	3	131,327.8	888.9	21.6	0.216
	Within groups	192	619,454.1	3226.3	78.4	
STRUCTURE groups at K = 4	Among predicted groups	4	161,762.3	1010.5	24.7	0.247
	Within groups	191	589,019.5	3083.9	75.3	
Eight wheat classes	Among wheat classes	7	139,984.3	813.6	20.0	0.200
	Within groups	188	610,797.6	3248.9	80.0	
Six breeding periods	Among six breeding periods	5	33,152.2	98.8	2.6	0.026
	Within groups	188	710,193.1	3777.6	97.5	
Four breeding periods	Among 4 breeding periods	3	27,653.3	118.3	3.1	0.031
	Within groups	190	715,692.0	3766.8	97.0	
Six breeding programs	Among 6 breeding programs	5	30,563.9	93.3	2.5	0.025
	Within groups	168	622,755.7	3706.9	97.6	
**SNPs under selection**						
STRUCTURE groups at K = 3 (524 SNPs)	Among predicted groups	3	8556.9	61.6	58.7	0.587
	Within groups	192	8315.2	43.3	41.3	
Eight wheat classes (1520 SNPs)	Among wheat classes	7	19,877.7	132.8	55.8	0.558
	Within groups	188	19,758.5	105.1	44.2	
Six breeding periods	Among six breeding periods	5	8242.4	47.7	14.9	0.149
	Within groups	188	51,010.9	271.3	85.1	
Four breeding periods	Among six breeding periods	3	7437.9	47.9	14.9	0.149
	Within groups	190	51,815.3	272.7	85.1	

For comparison purpose, the results computed from the 524, 1520, and 2314 outlier SNPs associated with the three predicted groups, 8 wheat classes, and six breeding periods, respectively are included.

### Diversity indices

**Figure 4:**
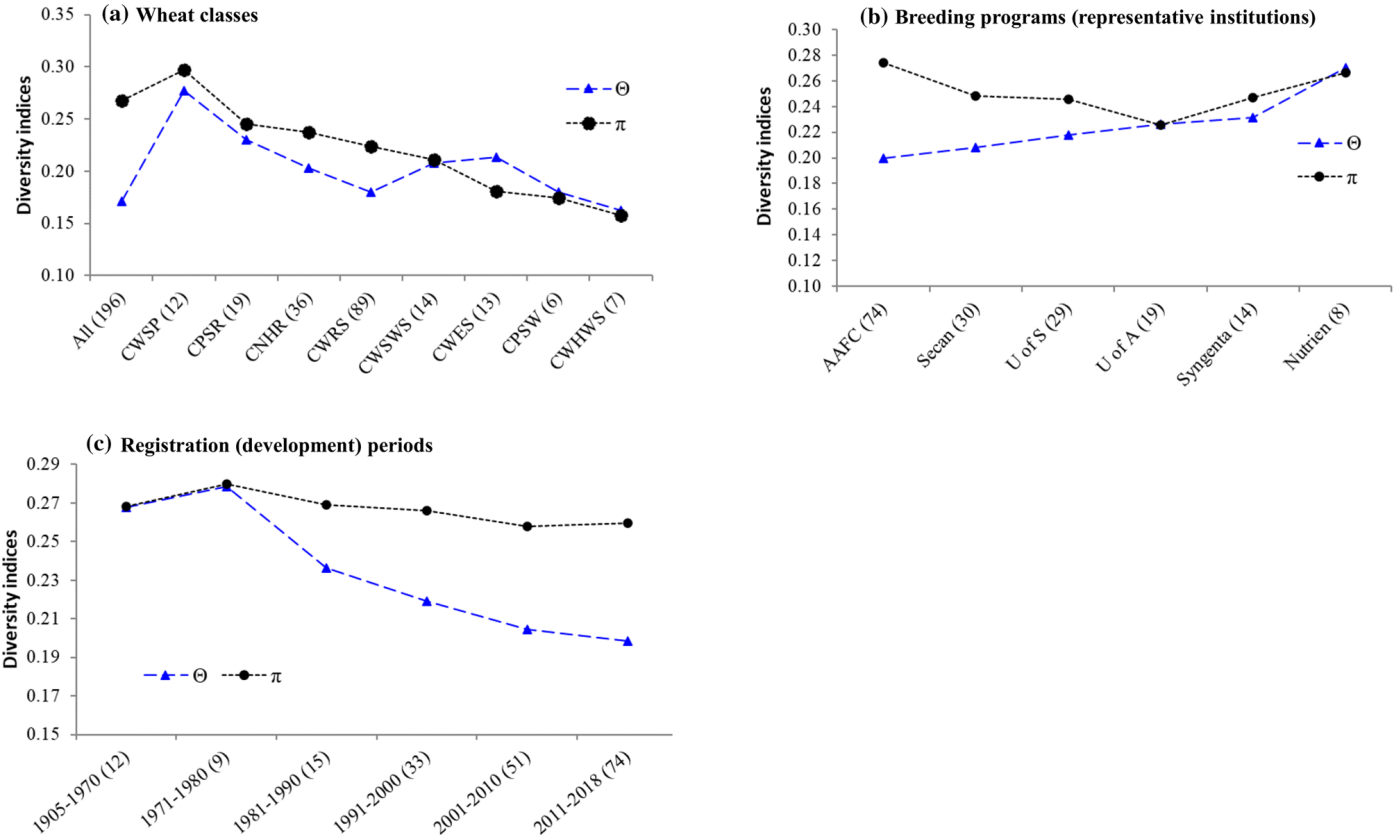
Comparison of diversity indices among (**a**) wheat classes, (**b**) registration or breeding periods, and (**c**) breeding programs. Sample sizes (N) are shown within brackets.

Supplementary Table S5 summarizes the proportion of segregating SNPs (Ps), theta (ϴ_S_), and nucleotide diversity (π) values computed for the different categorical variables. When all 196 cultivars were used for analyses, Ps, θ and π were 1.0, 0.171, and 0.267, respectively. We then compared these three diversity indices by assigning cultivars into different categorical variables. Of the 28,798 SNPs that were polymorphic across the 196 cultivars, the proportion of segregating SNP among the eight wheat classes varied from 39.7% in the CWHWS to 91.1% in the CWRS, with an overall average of 69.1%. θ and π values across the eight wheat classes varied from 0.162 to 0.278 and from 0.158 to 0.297, respectively. For both parameters, the lowest and highest values were observed within the CWHWS and CWSP classes, respectively. Varieties registered between 1971–1980 had similar θ and π values. Both parameters decreased since the 1980s until they reached the lowest among cultivars registered from 2011 to 2018 (Fig. 4). However, there was a much sharper decline in θ than π. Cultivars developed by the Wheat breeding program of the University of Alberta and Nutrien Ag Solutions Inc. gave similar θ and π values. Cultivars developed/registered by the Agriculture and Agri-Food Canada, Secan Association, and the University of Saskatchewan showed higher π and lower θ values. The converse was true for θ with varieties registered/developed by AAFC that had shown the highest and lowest proportion of polymorphic sites and θ, respectively, while those registered by Nutrien AG Solution Inc. (https://www.nutrienagsolutions.ca/) with the lowest polymorphism but the highest θ.

### Genetic distance and kinship

**Figure 5:**
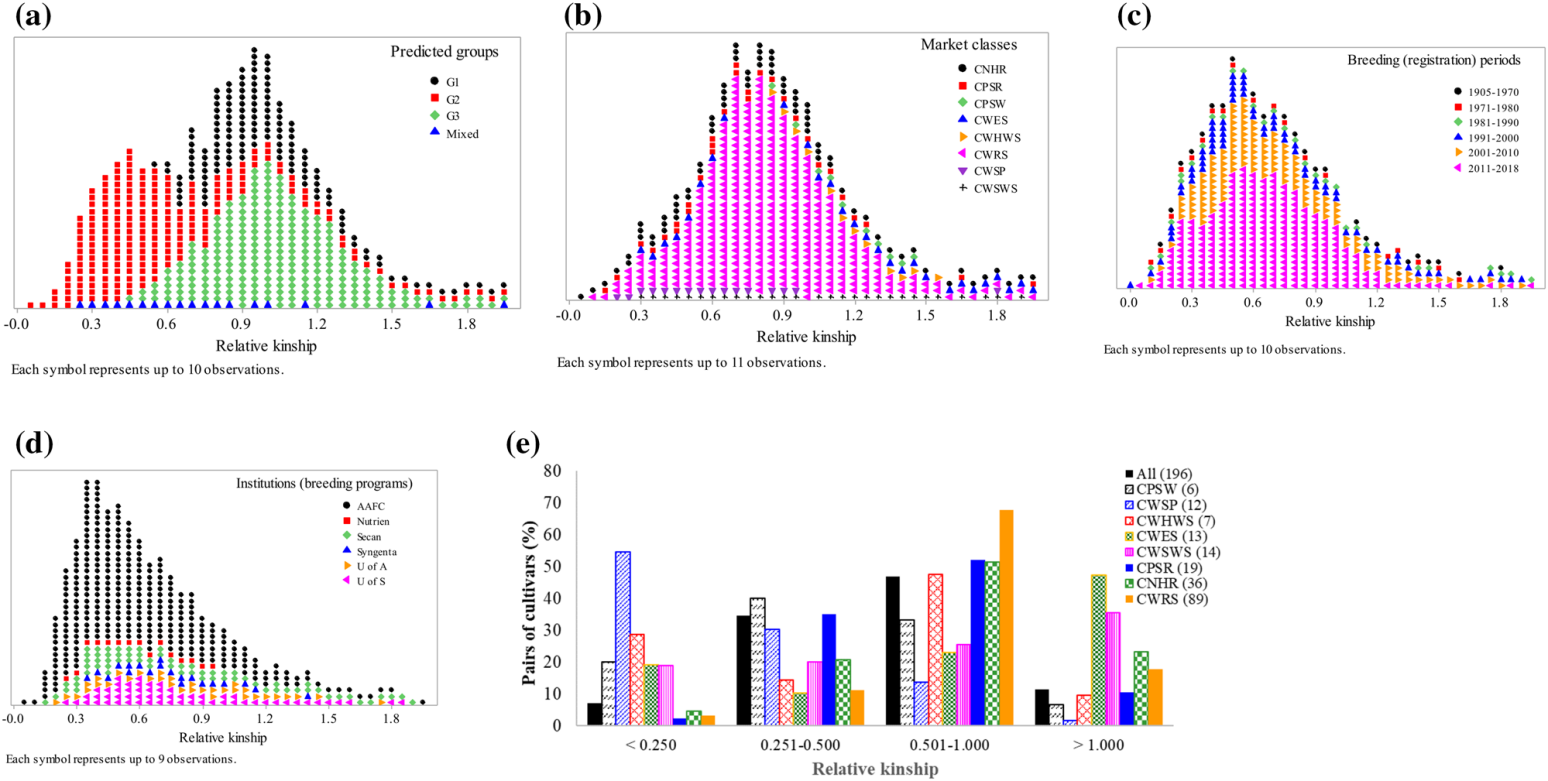
Frequency distribution of relative kinship values computed between pairs of 196 spring wheat cultivars based on 28,798 polymorphic SNPs. The plots were made by categorical variables with the y-axis indicating the number of pairs (**a**–**d**) and all pairs (**e**).

**Figure 6:**
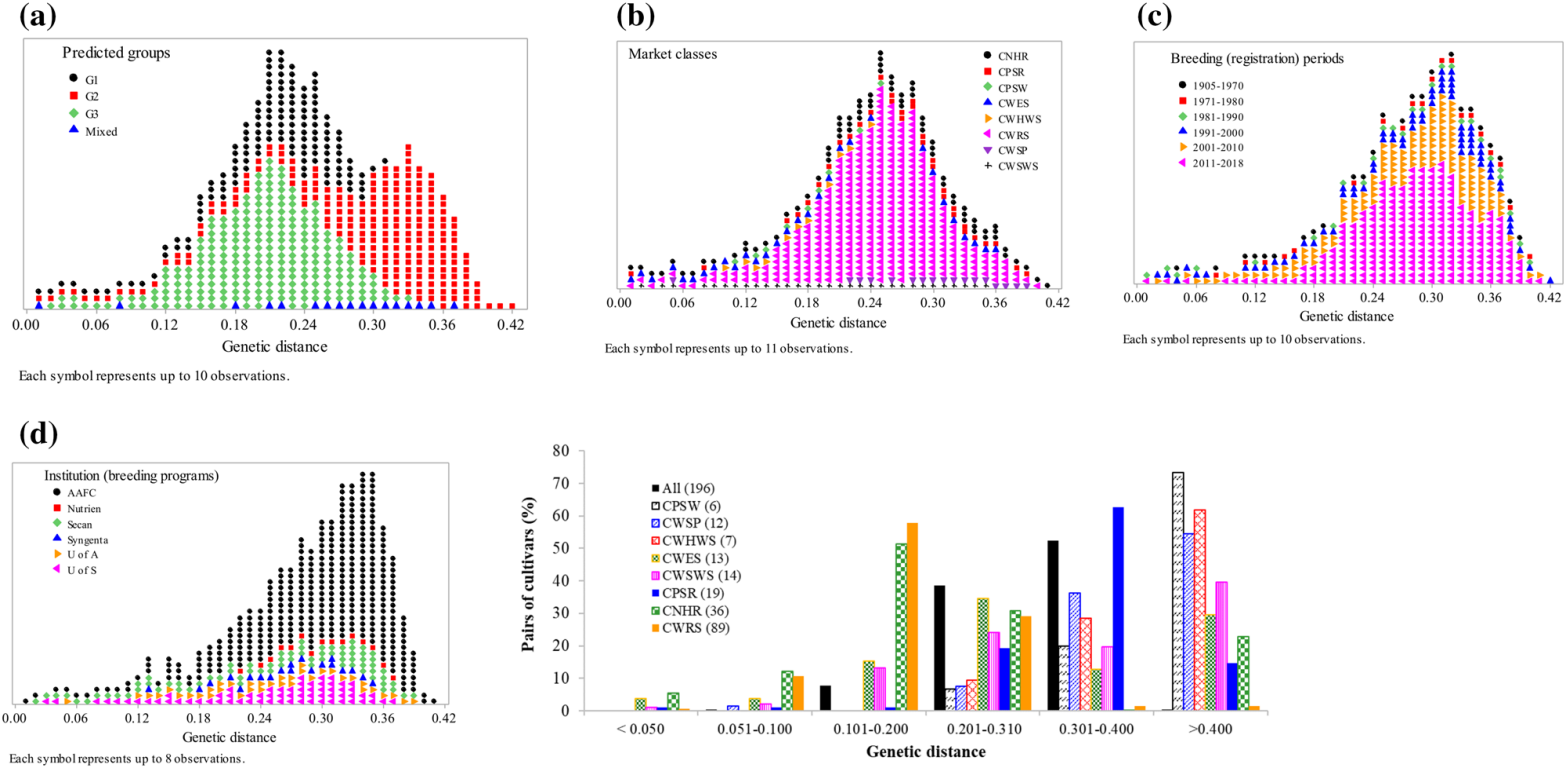
Frequency distribution of identity-by-state genetic distance computed between pairs of 196 spring wheat cultivars genotyped with 28,798 polymorphic SNPs. The plots were made by categorical variables with the y-axis indicating the number of pairs (**a**–**d**) and all pairs (**e**).

Kinship coefficients between pairs of the 196 cultivars varied from 0 between Springfield and AC Elsa to 1.97 between CDC NRG003 and AC Vista (on a scale of 0 to 2), with an average of 0.62 (Supplementary Table S6). About 7%, 35%, and 58% of the pairs had kinship values of < 0.250, 0.251–0.500, and 0.501–1.000, respectively (Fig. 5). Only ten pairs of cultivars had kinship values of < 0.05 and 58% of the pairs with kinship coefficients > 0.50. Kinship values among cultivars belonging to each wheat class were highly variable with 15.2% of the CWSP and 85.9% of the CWRS cultivars having kinship between 0.51 and 1.97. The genetic distance between pairs of the 196 cultivars varied from 0.007 between CDC NRG003 and AC Vista to 0.423 between Springfield and AC Elsa, with an overall average of 0.292 (Supplementary Table S3). As shown in Fig. 6, about 91% of pairwise distances ranged from 0.201 to 0.400. Sixty pairs of cultivars that accounted for 0.3% of the 19,110 pairwise comparisons had very low genetic distance and differed by just < 5% of the scored alleles, which all have high kinship values (1.77–1.97) and involved 55 cultivars. The eight cultivars that showed such a very low genetic distance and high kinship with five to eight other cultivars were AC Cora and Katepwa (8 pairs each), AC Michael, Conway, and Neepawa (7 pairs each), Benito and Kenyon (6 pairs each), and AC Minto (5 pairs). These eight cultivars are connected by a common parentage in their pedigree (see the discussion section). The remaining 47 cultivars showed very low genetic distance and high kinship with one or two cultivars. Nearly 27% of pairs of cultivars that were found to be highly similar originated from the SeCan (https://www.secan.com/) and 15% of them were from the AAFC.

We also compared genetic distance among pairs of cultivars belonging to each wheat class, which is summarized in Fig. 6 and Supplementary Table S3. None of the pairs of cultivars belonging to the CPSW, CWHWS, and CWSP showed a genetic distance below 0.05 (i.e., 5% of the scored alleles). In the other classes, we found some pairs differing by < 5% of the scored alleles, which included AC Phil and AC Reed (0.012) in CWSWS; both AAC Crossfield versus AAC Entice (0.012) and HY320 versus Biggar (0.012) in CPSR; Laser versus Wildcat (0.013), Glenlea versus AC Karma (0.030), and Glenlea versus Genesis (0.048) in CWES class. In both CNHR and CWRS classes, we found multiple pairs of cultivars that differed by < 5% of the scored alleles. Overall, the CWRS and CPSW classes showed the least and most genetically distant cultivars, respectively (Fig. 6). About 30% of the pairs of CWRS cultivars differed by more than 31% of the scored alleles as compared with 91–93% of the pairs of cultivars within CPSW, CWSP, and CWHWS classes.

### Detection of loci under selection

Using the hierarchical island model, the threshold F_ST_ values for declaring SNPs under selection based on three predicted groups at K = 3, the eight wheat classes, and six breeding periods were 0.22, 0.21, and 0.03 respectively. Using these threshold values, we found a total of 524, 1520, and 2314 SNPs with significantly (*p* < 0.05) different F_ST_ values among the three predicted groups, eight wheat classes, and six breeding periods, respectively (Supplementary Table S7). The selected outlier SNPs were distributed across ten chromosomes (1A, 1B, 1D, 2A, 2B, 2D, 4B, 5A, 5B, and 5D) and were primarily responsible for the observed molecular variance and genetic differentiation across the predicted groups and wheat classes (Table 2). Overall, the hierarchical island model identified a total of 3980 outlier SNPs of which 364 SNPs were identified twice and 7 SNPs thrice, and the remaining only once. A search for candidate genes around the 524, 1520, and 2314 outlier SNPs identified 13, 7, and 9 genes, respectively, with known effects on wheat phenotype. Some of the genes were detected using one of the three options while others using two options. Using all these three options, we found a total of 19 known genes across ten wheat chromosomes: *Gli-A3* (at 3.85–4.03 Mb), *Pm3b (4.49 Mb)*, *Glu-A3* (4.03–5.19 Mb depending on the version of physical maps), *Yr10* (6.16 Mb) and *Glu-A1* (510.21 Mb) on chromosome 1A; *Gli-B1* (4.97–5.00 Mb) and *Glu-B3* (6.89 Mb) on 1B; *Glu-D1* (414.59 Mb) on 1D; *Ppd-A1* (41.5 Mb) and *Ptr ToxA* (185.12 Mb) on 2A; *Ppd-B1* (63.35–63.47 Mb) and *Glu1B* (782.5–783.2 Mb) on 2B; *Ppd-D1* (at 36.21 Mb) and *GLU1C* (648.24 Mb) on 2D; *Rht-B1* (30.86–33.62 Mb) on 4B; *Vrn-A1* (587.41–590.40 Mb) and *Vrn-A2* (699.47–700.48 Mb) on 5A; *Vrn-B1* (573.80–577.20 Mb) on 5B, and *Vrn-D1* (467.18–470.05 Mb) on 5D (Supplementary Table S7).

Using the RAiSD outlier detection method and a chromosome-wise threshold (Supplementary Fig. S5) without any a priori know categorical variables, we identified a total of 41 genomic regions that have undergone selection (candidate selective sweeps) across all the 21 wheat chromosomes, with each chromosome harboring up to three selective sweeps (Supplementary Table S8). Each selective sweep region spans from 0.2 to 18.6 Mb and harbored between one and one hundred thirteen candidate genes (Table 3). To minimize the length of the paper, we briefly described only selective sweeps that map close to known genes controlling relevant traits. We identified a selective sweep on chromosome 1A that spans 0.34 Mb from 6.74 to 7.08 Mb, which consisted of eight candidate genes, including protein kinase domain (TraesCS1A02G011900, TraesCS1A02G012000, and TraesCS1A02G012100), RING-type domain-containing protein (TraesCS1A02G011700), and formin-like protein (TraesCS1A02G011800). The second selective sweep on chromosome 1A spans 0.51 Mb between 489.76 and 490.27 Mb and harbors four candidate genes, including Lipase_GDSL domain-containing protein (TraesCS1A02G295000). The selective sweep identified on 4A spans 18.59 Mb and harbors 23 candidate genes, including oxidoreductase (TraesCS4D02G161200) that plays a role in oxidation–reduction process, NADH dehydrogenase (TraesCS4D02G161400), Ribosomal_S7 domain-containing protein (TraesCS4D02G161500), Phosphatidylinositol 4-phosphate 5-kinase (TraesCS4D02G161800), and 1-phosphatidylinositol-4-phosphate 5-kinase (TraesCS4D02G161900).

**Table 3:** Summary of the 41 selective sweep regions identified using RAiSD.

Chrom	Selective sweep regions	Start position (bp)	End position (bp)	Interval (Mb)	No. of candidate genes
1A	1A:6741620-7084061	6,741,620	7,084,061	0.34	8
1A	1A:489755006-490268668	489,755,006	490,268,668	0.51	4
1B	1B:262133127-265500772	262,133,127	265,500,772	3.37	17
1B	1B:270792786-273404429	270,792,786	273,404,429	2.61	6
1B	1B:283713547-285363006	283,713,547	285,363,006	1.65	3
1D	1D:150206626-155199229	150,206,626	155,199,229	4.99	4
1D	1D:451838964-452086122	451,838,964	452,086,122	0.25	6
2A	2A:145990257-146379625	145,990,257	146,379,625	0.39	3
2A	2A:231417582-236635113	231,417,582	236,635,113	5.22	10
2A	2A:622576613-623355349	622,576,613	623,355,349	0.78	7
2B	2B:283064519-289677737	283,064,519	289,677,737	6.61	6
2D	2D:265636416-273096409	265,636,416	273,096,409	7.46	6
3A	3A:248231282-259046312	248,231,282	259,046,312	10.82	6
3A	3A:503615171-503988103	503,615,171	503,988,103	0.37	6
3B	3B:314095703-315418012	314,095,703	315,418,012	1.32	2
3B	3B:326740282-337814619	326,740,282	337,814,619	11.07	15
3D	3D:124715796-129988415	124,715,796	129,988,415	5.27	26
3D	3D:213737216-220665191	213,737,216	220,665,191	6.93	7
3D	3D:241816975-245127689	241,816,975	245,127,689	3.31	3
4A	4A:346859907-365454725	346,859,907	365,454,725	18.59	23
4B	4B:30337113-34227082	30,337,113	34,227,082	3.89	44
4B	4B:452331608-462660145	452,331,608	462,660,145	10.33	62
4D	4D:236718150-244723056	236,718,150	244,723,056	8.00	9
5A	5A:573275736-574053263	573,275,736	574,053,263	0.78	11
5A	5A:697821512-705031313	697,821,512	705,031,313	7.21	113
5B	5B:87295191-99114106	87,295,191	99,114,106	11.82	70
5B	5B:221414216-222502349	221,414,216	222,502,349	1.09	6
5B	5B:255481138-265608523	255,481,138	265,608,523	10.13	49
5D	5D:228875398-233752267	228,875,398	233,752,267	4.88	46
5D	5D:246645139-253259743	246,645,139	253,259,743	6.61	37
6A	6A:107534467-107781133	107,534,467	107,781,133	0.25	3
6A	6A:270087660-283284316	270,087,660	283,284,316	13.20	22
6B	6B:98756328-109469647	98,756,328	109,469,647	10.71	28
6D	6D:120017313-125631054	120,017,313	125,631,054	5.61	44
6D	6D:154596070-160775902	154,596,070	160,775,902	6.18	41
7A	7A:361090422-371085384	361,090,422	371,085,384	9.99	10
7A	7A:388356091-391001816	388,356,091	391,001,816	2.65	1
7A	7A:398571530-400702809	398,571,530	400,702,809	2.13	4
7B	7B:294347237-306561306	294,347,237	306,561,306	12.21	20
7D	7D:311163774-329143042	311,163,774	329,143,042	17.98	21
7D	7D:347694088-351950664	347,694,088	351,950,664	4.26	7

See Supplementary Table S8 for details.

One of the selective sweeps on chromosome 4B is located between 30.3 and 34.2 Mb and harbors a cluster of 44 candidate genes, including *Rht-B1* (TraesCS4B02G043100). The physical position of *Rht-B1* differed depending on the version of the physical maps, which corresponds to 30.86 Mb on the IWGSC RefSeq v1.0 and at 33.62 Mb on RefSeq v2.0. A selective sweep on chromosome 5A was located from 573.28 to 574.05 Mb and harbors eleven candidate genes, including NPH3 domain-containing protein (TraesCS5A02G375500), AA_permease_C domain-containing protein (TraesCS5A02G375600), and Aspartic peptidase (TraesCS5A02G376300). The second selective sweep on 5A was located between 697.82 and 705.03 Mb, which harbors 113 candidate genes, including the vernalization response *Vrn-A2* gene at 700.48 Mb. On chromosome 5B, we identified three selective sweeps located from 87.30 to 99.11 Mb, from 221.41 to 222.50 Mb, and from 255.48 to 265.61 Mb, which harbors seventy, six, and forty-nine candidate genes, respectively. Protein kinase domain-containing protein (TraesCS5B02G079200 and TraesCS5B02G080100), F-box domain-containing protein (TraesCS5B02G079500 and TraesCS5B02G079700), Dirigent protein (TraesCS5B02G080000), Hexosyltransferase (TraesCS5B02G080300), and RNA helicase (TraesCS5B02G080500) are among some of the candidate genes that fell within the 87.30–99.11 Mb interval. The selective sweep on 5B located between 221.41 and 222.50 Mb harbors six candidate genes, including acyl-[acyl-carrier-protein] desaturase (TraesCS5B02G123200), and Glycos transferase domain-containing protein (TraesCS5B02G123400).

One of the selective sweeps on chromosome 6A (6A:107534467-107781133) spans 0.25 Mb and harbors three candidate genes, including peroxidase (TraesCS6A02G136500) that plays a role in response to environmental stress, oxidation of toxic reductants and their removal, biosynthesis, and degradation of lignin, suberization, and auxin catabolism. On chromosome 7A, we detected three selective sweeps of which one region (7A:361090422-371085384) harbors ten candidate genes. Some of the candidate genes include Superoxide dismutase (TraesCS7A02G292100) that destroys radicals that are produced within the cells and toxic to biological systems, an intracellular protein transport (TraesCS7A02G292400) that is required for vesicular transport between the endoplasmic reticulum and the Golgi apparatus, transmembrane ascorbate ferrireductase 2 (TraesCS7A02G292700), and protein kinase domain-containing protein (TraesCS7A02G292800). The other selective sweep on 7A spans 2.13 Mb and harbors four candidate genes, including Diacylglycerol kinase (TraesCS7A02G297200), chloramphenicol acetyltransferase-like domain (TraesCS7A02G297300). On chromosome 7D, we identified two selective sweep regions at 311.16–329.14 Mb and 347.69–351.95 Mb, which harbored 21 and 7 candidate genes, respectively. The latter region consisted of P-loop containing nucleoside triphosphate hydrolase (TraesCS7D02G291100), a protein associated with somatic embryogenesis receptor kinases (TraesCS7D02G291200) that play roles in brassinosteroid signaling and regulation of plant architecture, KH type-2 domain-containing protein (TraesCS7D02G291300), P-loop containing nucleoside triphosphate hydrolase (TraesCS7D02G291500), diacylglycerol kinase (TraesCS7D02G291600), and chloramphenicol acetyltransferase-like domain superfamily (TraesCS7D02G291900).

### Linkage disequilibrium

**Figure 7:**
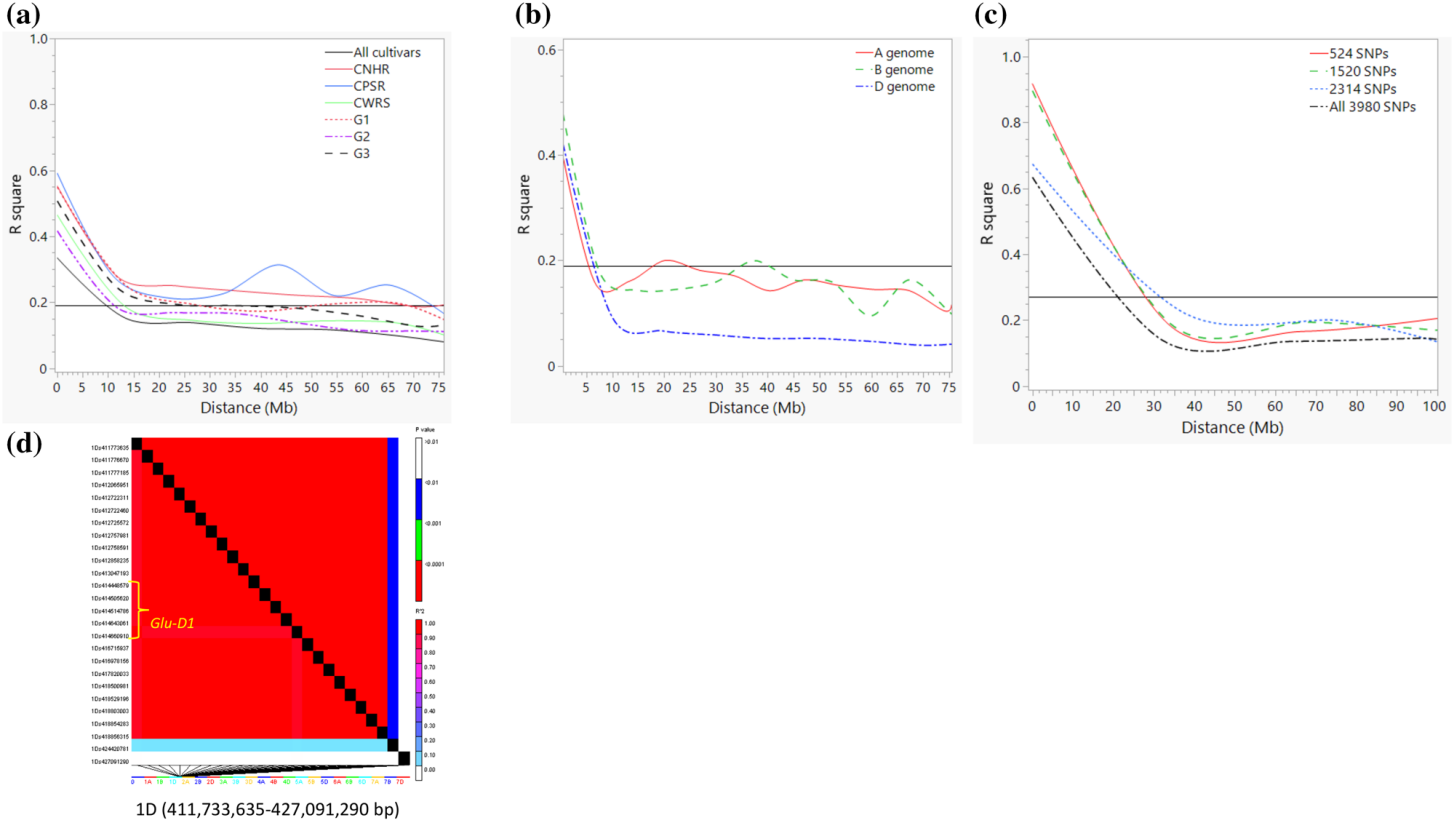
(**a**) Genome-wide linkage disequilibrium (LD) decay between pairs of 28,798 SNPs across all 196 cultivars, three predicted groups (G1, G2, and G3), and three wheat classes (CNHR, CPSR, and CWRS) that have 20 or more cultivars. The horizontal line shows the average squared coefficient of correlation (r^2^ = 0.19). Genome-wide LD fell below the average at 10 Mb. (**b**) Intra-chromosomal LD in the A-, B-, and D-genome based on 28,798 SNPs used for genotyping 196 cultivars. The horizontal line shows the average squared coefficient of correlation (r^2^) at r^2^ = 0.19. LD decay between 5 and 7 Mb depending on the genome. (**c**) Intra-chromosomal LD decay between pairs of outlier SNPs that have undergone selection among the three predicted groups (524 SNPs in red solid line), eight market classes (1520 SNPs in green dashed line), six registration or breeding periods (2314 SNPs in blue dotted line), and all 3,980 SNPs after excluding common markers(black broken line). (**d**) LD decay plot on chromosome 1D between 411.7 and 427.1 Mb to show SNPs with high LD around the *Glu-D1* gene at 414.6 Mb.

LD was calculated for nearly one million pairs from the 28,798 SNPs out of which 25.3% of the pairs had zero LD and 22% of the pairs showed r^2^ ≥ 0.19, which is the overall mean of all pairs. The latter includes 80 and 253,609 inter-and intra-chromosomal LD pairs, respectively. Genome-wide LD declined to 0.19 within 10 Mb (Fig. 7a), but it was faster for A-genome (5 Mb), B-genome (7 Mb), and D-genome (6 Mb) (Fig. 7b). LD decay was much slower within the wheat classes and predicted groups, which was due to the smaller number of cultivars in each group that in turn reduced the number of polymorphic SNPs for analyses. Overall, r^2^ values computed between pairs of the 524 outlier SNPs identified by the hierarchical island model for the three predicted groups and the 1520 SNPs for the eight wheat classes were much greater than those computed for the 2314 SNPs associated with breeding periods (Fig. 7c). Figure 7d shows an example of pairwise r^2^ values computed from SNPs identified through the hierarchical island model, which demonstrates a very strong LD on chromosomes 1D around the *Glu-D1* gene that maps from 414.6 Mb. Details results on LD and haplotype blocks will be presented as part of genome-wide association studies to map genomic regions associated with agronomic and end-use quality traits as well as resistance to five priority diseases.

## Discussion

### Genetic diversity and population structure

Knowledge of relative kinship coefficients and population structure is essential for multiple purposes, including understanding the degree of relatedness in the germplasm, monitoring the pedigree structure of progenies, correctly mapping the association between molecular markers and phenotypic traits, and assessing the accuracy of different genomic selection models^48–52^. Although several of the cultivars used in the present study shared one or more common parents, nearly 52% and 39% of the pairs differed by 30–40% and 20–30% of the scored alleles, respectively (Supplementary Table S3), suggesting large genetic variation among most Canadian western spring wheat cultivars. Our results agree with a previous study that used 81 spring wheat cultivars^29^. Seventy-two of the 196 cultivars were common between the two studies. Relative kinship coefficients, however, revealed the presence of a very strong genetic relationship among most pairs of cultivars. As described in other studies^48,53^, we expected to find kinship values close to 0 among unrelated cultivars (varieties), < 0.25 for half-sib, 0.5–1.0 for full-sib and > 1.0 highly similar cultivars. We found only ten pairs of cultivars with kinship coefficients below 0.05, 7.1% of the pairs with kinship values ranging from 0.05 to 0.25, 34.6% with kinship ranging from 0.251 to 0.500, and the remaining 58.3% of the pairs with greater than 0.50 kinship (Fig. 5, Supplementary Table S5). Hence, our kinship values demonstrate the presence of very strong genetic relationship among most pairs of cultivars, suggesting a substantial redundancy in genomic composition that is likely due to the repeated use of a few popular varieties as parents of multiple cultivars, which is in agreement with a previous study in wheat^54^. Using a total of 270 spring wheat cultivars released in western Canada (47), USA (133), and Mexico 90), the former authors reported lower genetic diversity and high coefficients of parentage among cultivars belonging to the CWRS class, which is comparable to a group of full sibs or sister cultivars. One of the reasons cited by the authors was the adoption of a rigid classification and quality standard, which precluded breeders from incorporating diverse sources of genetic diversity using different breeding methods other than the backcrossing schemes.

We compared the kinship and genetic distance values for a few representative spring wheat varieties. AAC Elie^55^ and AAC Brandon^56^ differed by just 1.1% of the alleles of the 28,798 SNPs (Supplementary Table S3) with a very high kinship value (1.95) (Supplementary Table S6). Both cultivars belong to the CWRS class and were developed by the same team at the Agriculture and Agri-Food Canada, Swift Current Research and Development Centre in Saskatchewan from the same cross (Superb/CDC Osler//ND744) made in 2003. They have also similar phenotypic characteristics, including awned spike, low lodging score, a short plant stature, grain yield and time to maturity, and resistance to prevalent races of leaf and stem rusts, intermediate resistance to Fusarium head blight, and loose smut^55,56^. AC Cora showed high kinship coefficients (1.77–1.89) and very low genetic distance (0.023–0.049) with Katepwa, Benito, AC Michael, AC Minto, Katepwa, Kenyon, and Alikat. These eight cultivars are linked by common parents. AC Cora and AC Minto were derived from Katepwa/RL4509^57^ and Columbus/BW63//Katepwa/BW552^58^, respectively, with Katepwa selected from Neepawa*6lRL2938/3/Neepawa*6//CI8154/2 *Frocor^59^. AC Michael, Kenyon, and Columbus were selected from Park/Neepawa^60^, Neepawa*5/Buck Manantial^61^, and Neepawa*6/RL 4131^62^, respectively.

Carberry is a doubled haploid cultivar derived from a cross between Alsen and Superb^63^. The kinship value between Carberry and Superb was 1.52 (Supplementary Table S5). AC Barrie differed from Neepawa and Columbus by 15.6% and 14.6% of the scored alleles, respectively, while Carberry differed from Superb by 10.2% of the scored alleles (Supplementary Table S3). The Canadian wheat breeding programs have been somewhat closed within each class, with little to no introgression of distant germplasm to maintain the end-use quality traits required by the Canadian Grain Commission. The correlations between pedigree and marker-based kinship range from 0.21^64^ to 0.71^65^ depending on the sample size, marker type, and marker density. The highest correlation was found by Fradgley and colleagues who reported a correlation coefficient of 0.71 based on 409 diverse wheat varieties genotyped with 4009 SNPs^65^. Different studies have emphasized the need for a large number of polymorphic markers to obtain reliable estimates for the relatedness of individuals^66^. Overall, our relative kinship and genetic distance matrices among the pairwise comparisons of the 196 spring wheat cultivars would be valuable for spring wheat breeders to aid in the selection of parents for future new breeding starts. Currently, the Canadian Food Inspection Agency (CFIA) is responsible for the registration of new varieties (cultivars) and demands the candidates possess a combination of 30-40 target phenotypic traits depending on the market class (https://grainscanada.gc.ca/en/grain-quality/grain-grading/wheat-classes.html). To avoid redundant registration of highly similar cultivars, we strongly suggest to include molecular markers data as part of the application package for the registration of new wheat cultivars in Canada.

Using phylogenetic analysis, PCA, and the model-based structure analyses, we examined the population structure of the cultivars across breeding periods, breeding programs, and wheat classes. All these methods revealed three major groups (Fig. 1 and Fig. 3). Cultivars in the CWRS class were divided into two groups, while those in the CNHR class were distributed across all three groups. The remaining five classes (CPSR, CPSW, CWES, CWSP, and CWSWS) formed a single group, which agrees with our previous study that used 81 spring wheat cultivars^29^, but we were not able to explain why the CWRS and CNHR cultivars were divided into 2–3 subgroups. Although individuals that cluster together to the same group likely shared ancestral cultivars in their pedigree, different studies have reported the lack of clear patterns of relationship based on wheat classes^67^.

Nearly half of the 28 pairwise comparisons of F_ST_ values showed very great genetic differentiation among wheat classes with only CWRS and CNHR classes showing very little genetic differentiation (F_ST_ < 0.05), which suggests a close relationship between these two classes. The moderate to a very great level of genetic differentiation among most pairs of the wheat classes (Supplementary Table S4) suggests that selection during wheat breeding has significantly altered allele frequencies across a large number of loci (Supplementary Fig. S5). It may also be due to the use of a small number of distinct founders (parental cultivars) to retain the end-use quality traits, which agrees with the pedigree information of several cultivars grown in the Canadian prairies^68^.

### Genetic diversity trend by categorical variables

We first examined the trend in genetic diversity indices across six breeding or registration periods, which revealed a substantial reduction both in θ and π values since the 1980s (Fig. 4). Both parameters were the highest among cultivars registered from 1970 to 1980 and then declined to the lowest from 2011 to 2018, which agrees with previous studies in Canadian spring wheat^1,4,12–14^ but disagree with the US wheat germplasm^10^. Using 370 SSR genotype data of 75 Canadian hard red spring wheat cultivars released from 1845 to 2004, Fu and Somers reported a significant net reduction in allelic diversity in every part of the wheat genome of older cultivars. They found out that about 38% and 44% of the alleles detected in older cultivars were retained and lost in newer cultivars, respectively, and 18% of the alleles detected in newer cultivars were not present in older cultivars, which resulted in 17% net reduction of the total SSR variation^14^. Although it is not practical to do similar comparisons between the biallelic SNPs and the multi-allelic SSR markers, both θ and π showed a continual decline in recent cultivars. θ is computed from the observed homozygosity, while π is computed from the mean number of pairwise differences^22,45^. Sthapit and colleagues studied the genetic diversity of 320 historical and modern spring and winter wheat varieties cultivated in the U.S. Pacific Northwest for over 120 years using 1370 polymorphic SNPs. They found no long-term shifts in genetic diversity both in spring and winter wheat but noted significant fluctuations within wheat classes and the most widely grown cultivars. In hard red spring wheat, for example, genetic diversity was high from 1970 to 1999 and then dropped below the level of diversity observed among cultivars registered before 1930. Hard red winter wheat diversity from 2000 to 2019 was higher than before 1930, whereas soft white spring and soft white winter had the same level of diversity from 2000 to 2019 as they were before 1930^10^. We discovered differences in both θ and π values across the eight wheat classes with cultivars belonging to CWHWS and CWSP showing the lowest and highest diversity indices, respectively (Fig. 4), which agrees with a recent study in US wheat germplasm described above^10^.

The inconsistent results reported in the literature regarding the impact of plant breeding on the genetic diversity of crops can be caused by several factors, including technical limitations associated with genetic diversity assessment, sample size, the type of molecular marker (dominant, codominant multiallelic, and biallelic), marker density and genome coverage, and the level of genetic purity of the germplasm. Many breeders still struggle to balance the level of heterogeneity in the germplasm (including retention of residual heterozygosity over generations to maximize diversity within a given cultivar) vis-a-vis the need in developing genetically uniform (pure) germplasm to meet the variety registration/release requirements and retaining end-use quality traits. The development of genetically pure cultivars with heterogeneity of < 1% would help breeders not only meeting the distinctness, stability, and uniformity requirements^69,70^, but also would be easier for seed production, developing reference fingerprints for variety identification and verification, and fetching premium price due to better quality. For such purpose, the use of molecular markers for selecting and maintaining genetically pure or nearly so advanced breeding lines plays a very critical role^71–73^, which has been implemented as a standard quality control/quality assurance method in most international plant breeding programs irrespective of mating systems. The development of genetically pure cultivars, however, significantly reduces within variety genetic diversity that may increase vulnerability to changing biotic (diseases, pests, and weeds) and abiotic (e.g., drought, soil fertility, soil acidity/alkalinity) stresses^74,75^.

### Role of loci under selection

Using the F_ST_-statistics based outlier detection method and a priori known categorical variables (three predicted groups based on the model-based population structure, the eight wheat classes, and six breeding periods), we identified from 524 to 2314 SNPs showing evidence of selection (Supplementary Table S7). Some of the genomic regions showing evidence of selection are physically close to genes contributing agronomically important phenotypes in wheat or other plants. The most obvious examples are the 19 genes with known phenotypic effects, which included the height reducing *Rht-B1*^76^, the stripe rust resistance *Yr10*^77^, the powdery mildew resistance *Pm3b*^78^, the Ptr ToxA host selective toxin^79^, the photoperiod response *Ppd-A1*, *Ppd-B1* and *Ppd-D1*^80^, the vernalization response *Vrn-A1*, *Vrn-A2*, *Vrn-B1*, *Vrn-D1*^80,81^, the glutenin strength due to *Glu-1* loci (*Glu-A1*, *Glu-D1, Glu1B*, and *Glu1C*) and *Glu-3* loci (*Glu-A3, Glu-B3*)^82–85^, and gliadin *Gli-B1*, *Gli-A3*^86,87^ genes. Similar results have been reported in other studies that identified several candidate selection regions containing genes that regulate diverse traits, including flowering time^7^.

Using selective sweep analysis in RAiSD, we uncovered 41 candidate selective sweep regions across all the 21 wheat chromosomes, with each region harboring between one and one hundred thirteen candidate genes (Table 3). Overall, we identified a total of 816 candidate genes that fell across the 41 selective sweep regions of which some genes have known phenotype effects. However, only *Rht-B1* and *Vrn-A2* were common among the known genes identified using both the F_ST_-based and RAiSD outlier detection methods. The F-statistics method detects loci under selection by examining the joint distribution of F_ST_ values and heterozygosity under a hierarchical island model without using any marker position. On the other hand, the RAiSD method computes μ statistics using a fixed-size SNP window for a significant reduction in genetic diversity and is highly dependent on marker density and physical information. It relies on three factors that account for the expected reduction of variation in the region of a sweep, the shift in the site frequency spectrum toward low- and high-frequency derived variants, and high LD on each side of a beneficial mutation and low LD between loci that are located on different sides of the beneficial allele^15^. Hence, results among methods differ substantially, which have been described in different studies^15,21,88–90^. Using the total variance F_ST_-based outlier detection method in Lositan^23^, the hierarchical island model in Arlequin^22^, the Bayesian genome scan method implemented in BayeScan^24^, N'Diaye et al.^88^ identified 403, 397, and 144 outliers out of the initial 4235 SNPs; however, only four SNPs were common in all three methods and 397 SNPs were common between Lositan and Arlequin. Crisci et al.^91^ and Alachiotis and Pavlidis^15^ compared SweepFinder, SweeD, and OmegaPlus and found highly variable results among these methods. Multiple factors affect the identification of SNPs and genomic regions under selection, including genotyping errors, marker density, allele frequencies, population structure, variation in mutation rate, and sensitivity to both false positives (Type I) and false negatives (Type II) error rates^89^. Combining results from two or more outlier detection methods could minimize the false positives and improve the reliability of the identified regions^92^. In our study, we think the outlier detection system based on the F_ST_-based hierarchical model is more convincing than the RAiSD, but the former method has been frequently criticized for its high Type I and Type II error rates^89,93^.

## Conclusion

We characterized the extent of molecular variation, population structure, and genetic differentiation of diverse historical and modern Canadian spring wheat cultivars and identified genomic regions that have undergone selection. Overall, the spring wheat cultivars were clustered into three groups with no distinct patterns of clustering across most wheat classes, breeding programs, and breeding periods. We observed a continuous decline in the genetic diversity indices since the 1980s until it reached its lowest estimates within cultivars registered from 2011 and 2018. Using two outlier detection methods, we identified several SNPs and/or selective sweeps that were physical close to known genes that regulate response to photoperiod (*Ppd-A1*, *Ppd-B1,* and *Ppd-D1*) and vernalization (*Vrn-A1*, *Vrn-A2*, *Vrn-B1*, *Vrn-D1*), glutenin strength (*Glu-A1*, *Glu-A3, Glu-B3*, *Glu-D1, Glu1B*, and *Glu1C*), gliadin (*Gli-B1*, *Gli-A3*), stripe rust resistance due to *Yr10*, powdery mildew resistance due to *Pm3b*, and insensitivity to Ptr ToxA host selective toxin. Further studies are needed to understand if the same regions would be detected using genomewide association studies and to identify haplotype blocks and their effect on different phenotypic traits. Our results provide valuable information and baseline data in spring wheat genetics and breeding studies, including understanding the degree of relatedness in the germplasm, selecting parents for new breeding starts, and mapping the association between molecular markers and phenotypic traits.

## Supplementary Information

Supplementary Figures.

Supplementary Table S1.

Supplementary Table S2.

Supplementary Table S3.

Supplementary Table S4.

Supplementary Table S5.

Supplementary Table S6.

Supplementary Table S7.

Supplementary Table S8.

## Data Availability

All relevant data are within the paper and its Supporting Information Files.
