# Supplementary material for: Genetic diversity and selective sweeps in historical and modern Canadian spring wheat cultivars using the 90K SNP array
Source: Sci Rep. 2021 Dec 10;11:23773. doi: 10.1038/s41598-021-02666-5 (PMC8664822; doi:10.1038/s41598-021-02666-5)
Supplement: Supplementary file 1 — Supplementary Figures. [file 41598_2021_2666_MOESM1_ESM.pdf]

Genetic diversity and selective sweeps in historical and modern Canadian spring wheat cultivars using the 90K SNP array

Kassa Semagn[1]*, Muhammad Iqbal[1], Nikolaos Alachiotis[2], Amidou N'Diaye[3], Curtis Pozniak[3], and Dean Spaner[1]*

[1]Department of Agricultural, Food and Nutritional Science, 4-10 Agriculture-Forestry Centre, University of Alberta, Edmonton, AB T6G 2P5, Canada.

[2]University of Twente, Faculty of Electrical Engineering, Mathematics and Computer Science, 3230 Enschede, OV, The Netherlands.

[3]Crop Development Centre and Department of Plant Sciences, University of Saskatchewan, 51 Campus Drive, Saskatoon, SK S7N 5A8, Canada.

*Corresponding authors: fentaye@ualberta.ca; dean.spaner@ualberta.ca

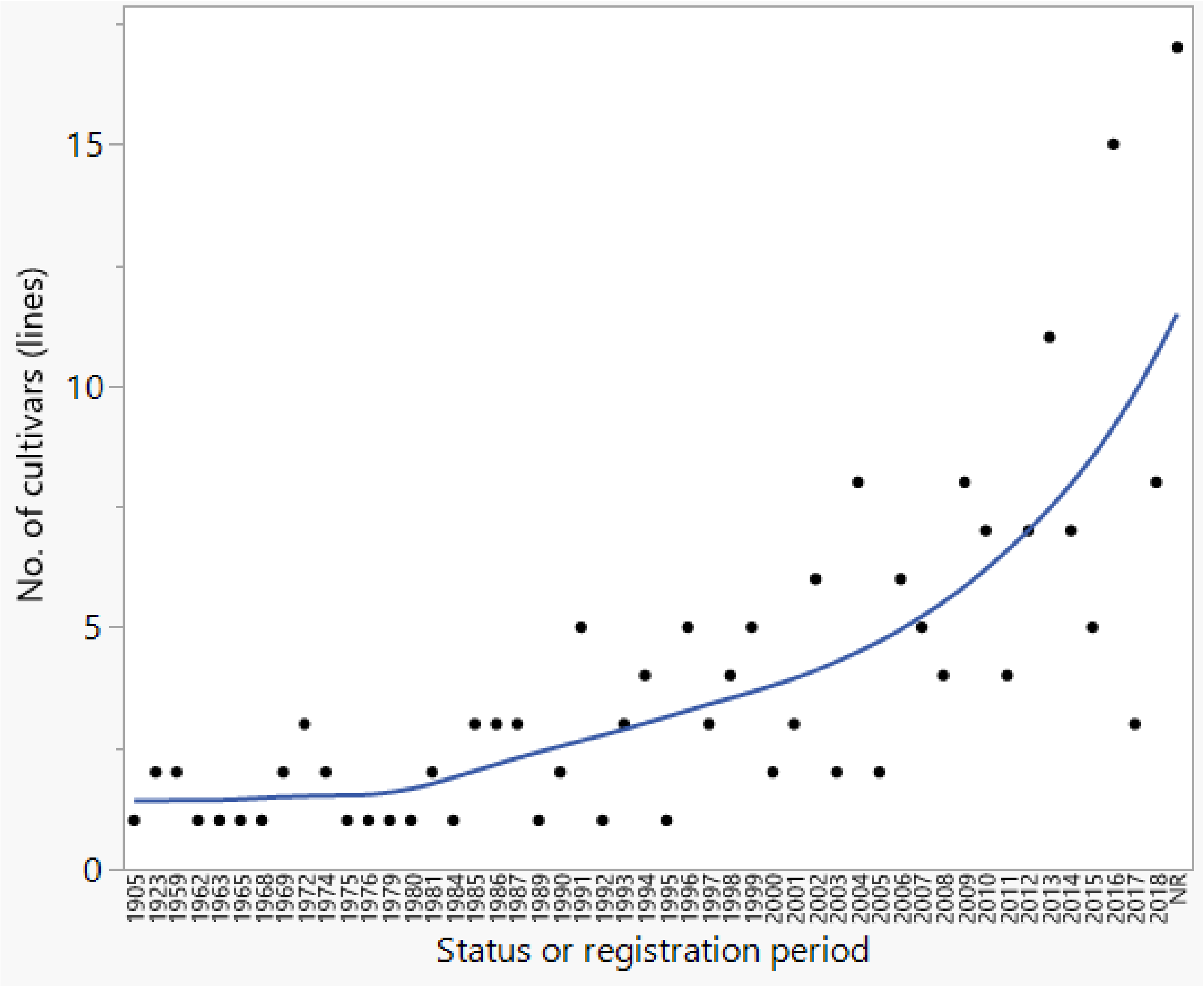

Supplementary Figure S1. Summary of the status of 196 Canadian spring wheat varieties and cultivars used in the present study. See Supplementary Table S1 for details of cultivars registered in each year. Unregistered cultivars are given under "NR".

Supplementary Figure S2. Population structure of 196 spring wheat cultivars based on 28,798 polymorphic SNPs: (a) plot of LnP(D) and an ad hoc statistic ΔK calculated for K ranging from 1 to 8, with each K repeated thrice; (b) population structure at K = 2, K = 3 and K = 4, with each accession represented by a single vertical line that is partitioned into K colored segments, with lengths proportional to the estimated probability membership value (y-axis); and (c) summary of genotypes belonging to each predicted group by wheat classes at K = 2, K = 3 and K = 4. See Supplementary Table S1 for details of genotypes belonging to each group.

(**a**) plot of LnP(D) and an ad hoc statistic ΔK

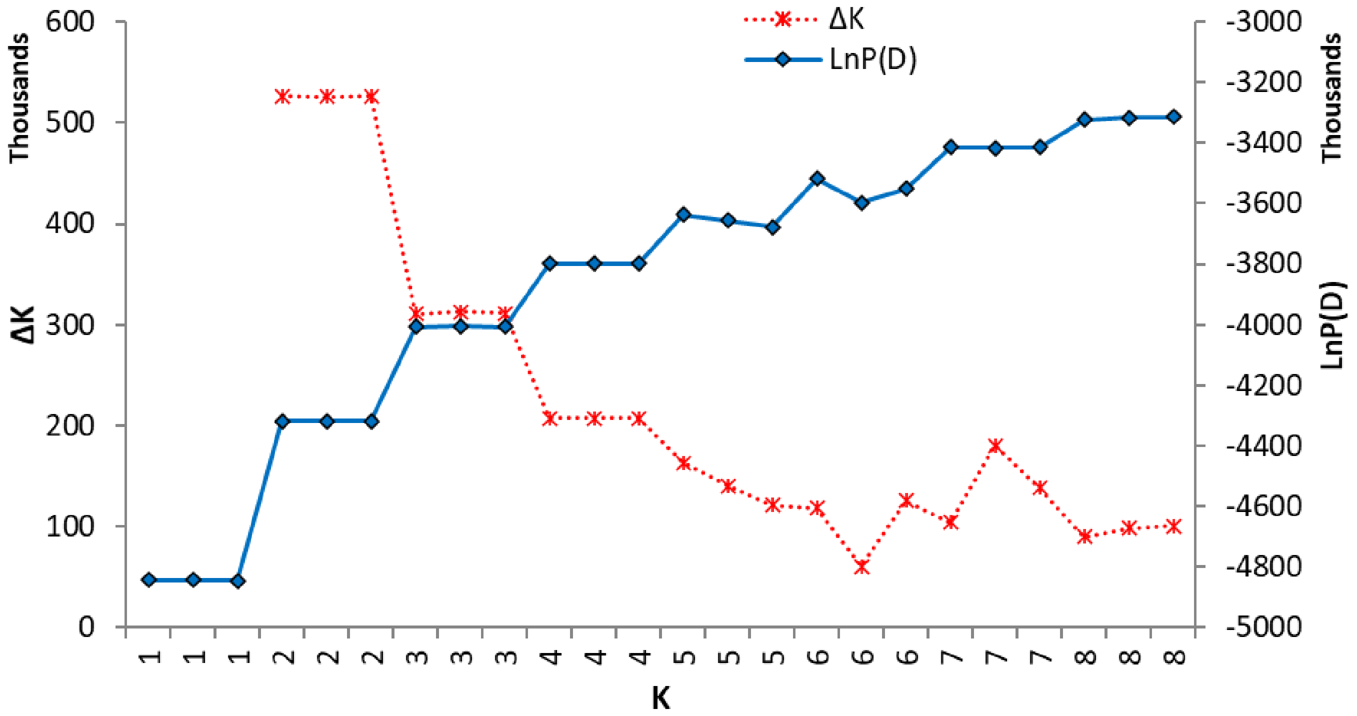

Figure S2

(b) population structure at K=2 Top), K=3 (middle) and K=4 (bottom)

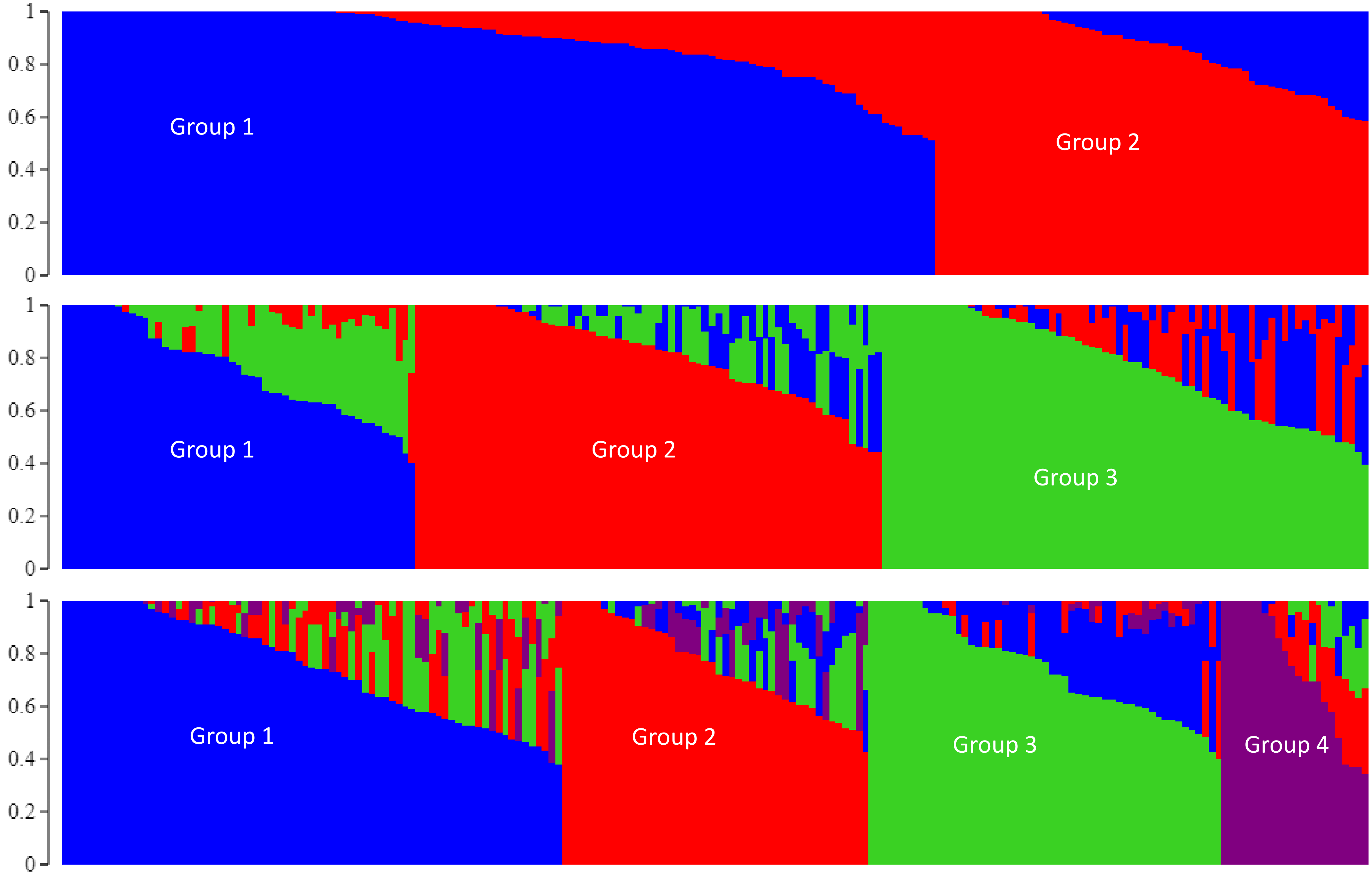

Figure S2 (continued)

(**c**) Summary of genotypes belonging to each predicted group by wheat classes at K = 2, K = 3 and K = 4

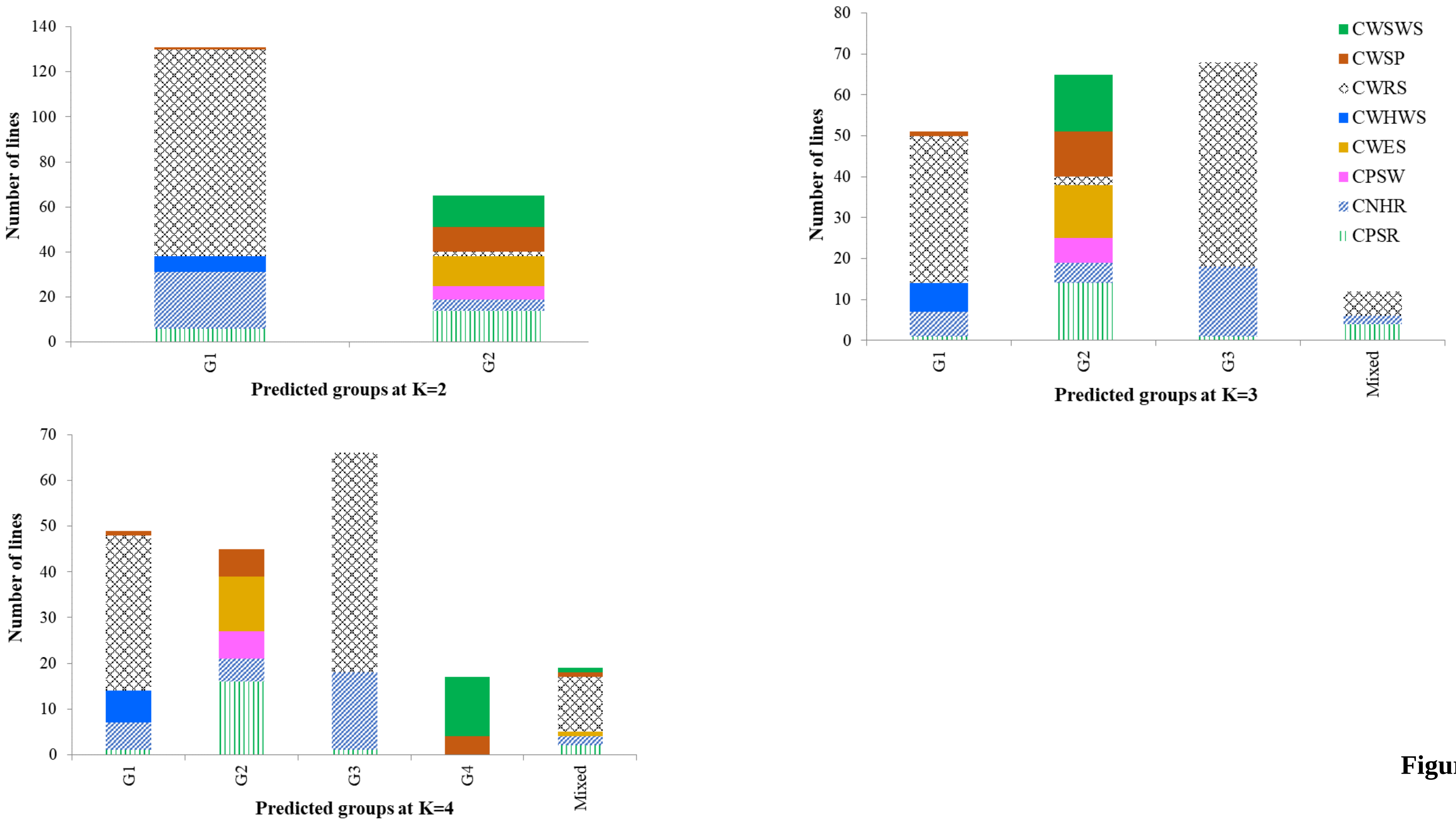

Figure S2 (continued)

Supplementary Figure S3. Plots of PC_1 (11.3% of variation), PC_2 (7.2%), and PC_3 (4.8%) from principal component analyses of 196 spring wheat cultivars genotyped with 28,798 polymorphic SNPs: Plots were made based on (a) eight wheat classes, d) six breeding periods, (c) four breeding periods, and (d) six breeding programs (representative institutions).

(**a**) Scatter plot based on eight wheat classes: CWRS (black), CNHR (blue), CPSR (red), CPSW (lime), CWES (olive), CWHWS (pink), CWSP (aqua), and CWSWS (orange). Most CNHR wheat clustered with CWRS.

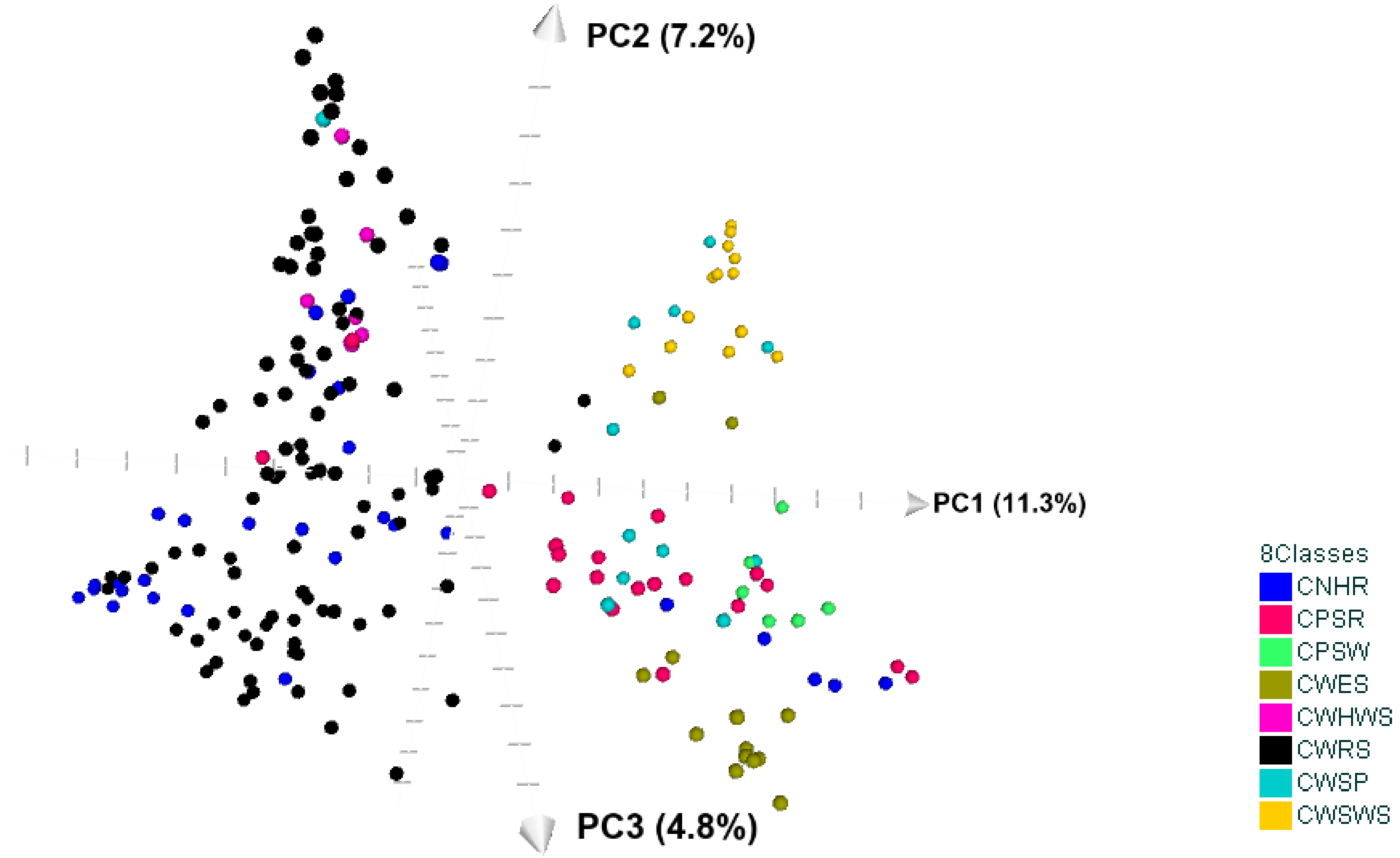

Figure S3 (continued)

(**b**) Scatter plot based on six breeding periods: 1905-1970 (blue), 1971-1980 (red), 1981-1990 (olive), 1991-2000 (green), 2001-2010 (purple), 2011-2018 (black), and (e) others (yellow).

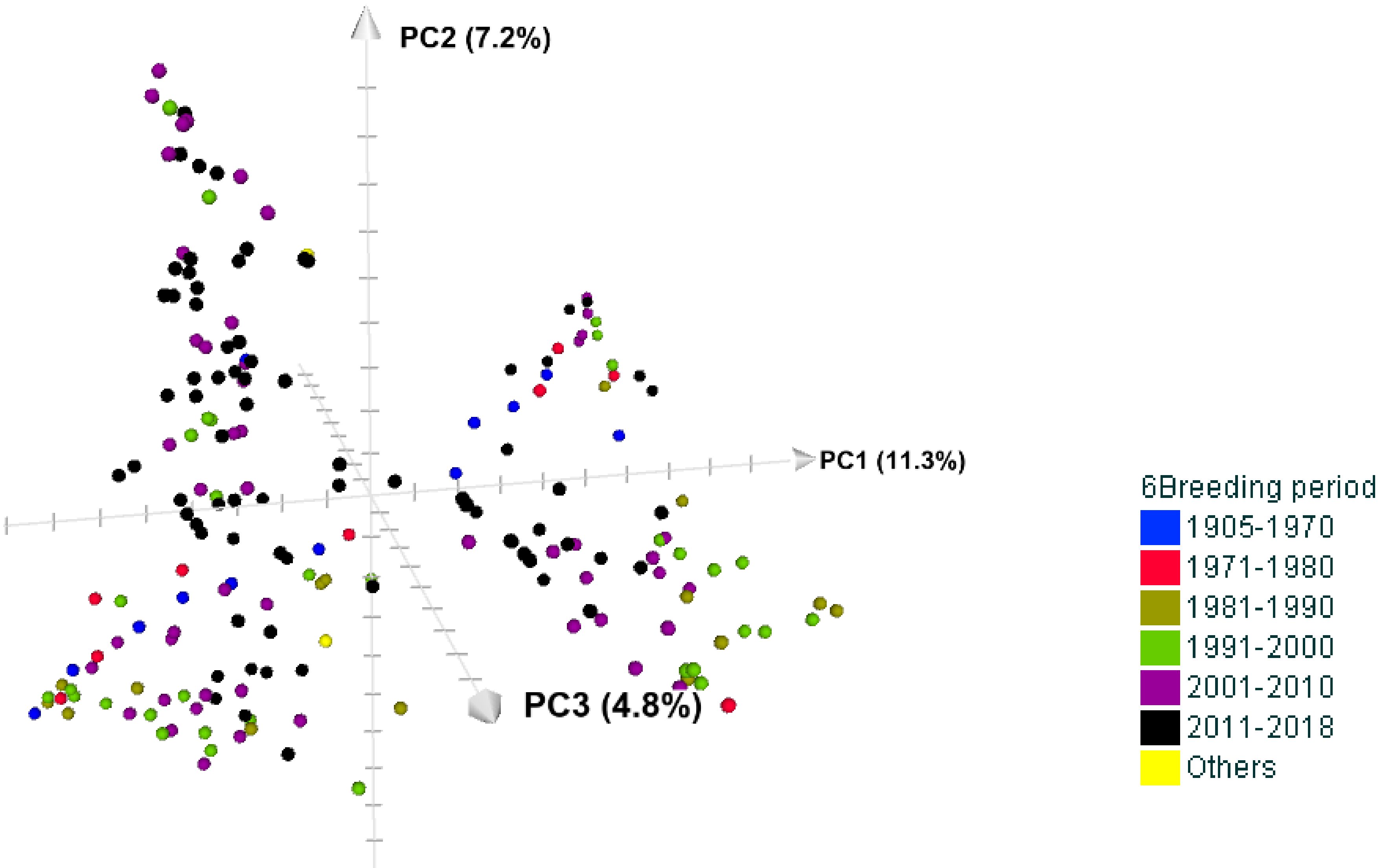

Figure S3 (continued)

(**c**) Scatter plot based on four breeding period: 1905-1970 (black), 1971-2000 (blue), 2001-2010 (red), 2011-2018 (olive), and others (yellow).

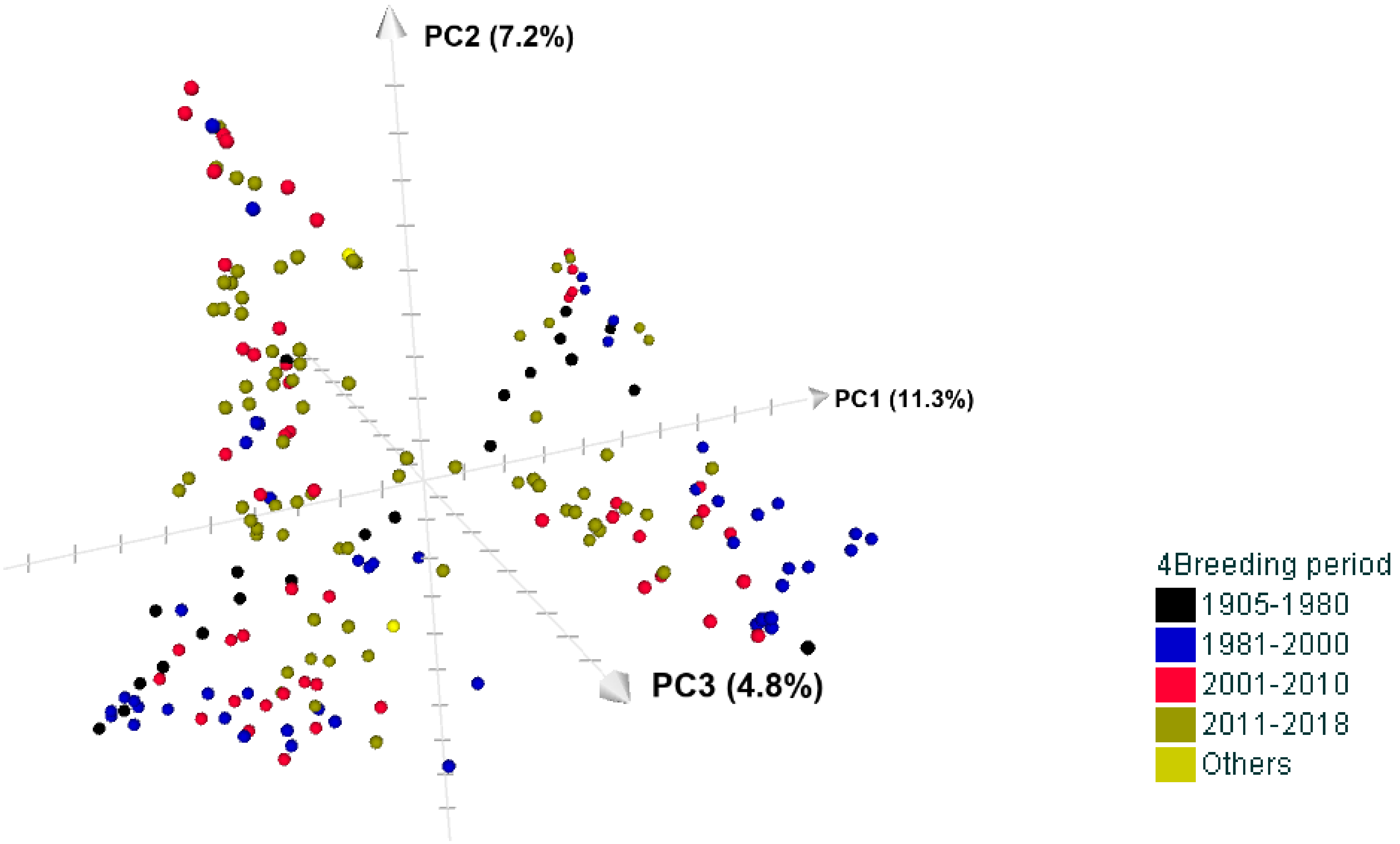

Figure S3 (continued)

(**d**) Scatter plot based on six breeding programs (representative institutions): Agriculture and Agri-Food Canada (black), University of Saskatchewan (aqua), University of Alberta (blue), Secan Association (red), Syngenta Canada Inc. (pink), and Nutrien AG Solutions Inc. (olive), and others (yellow).

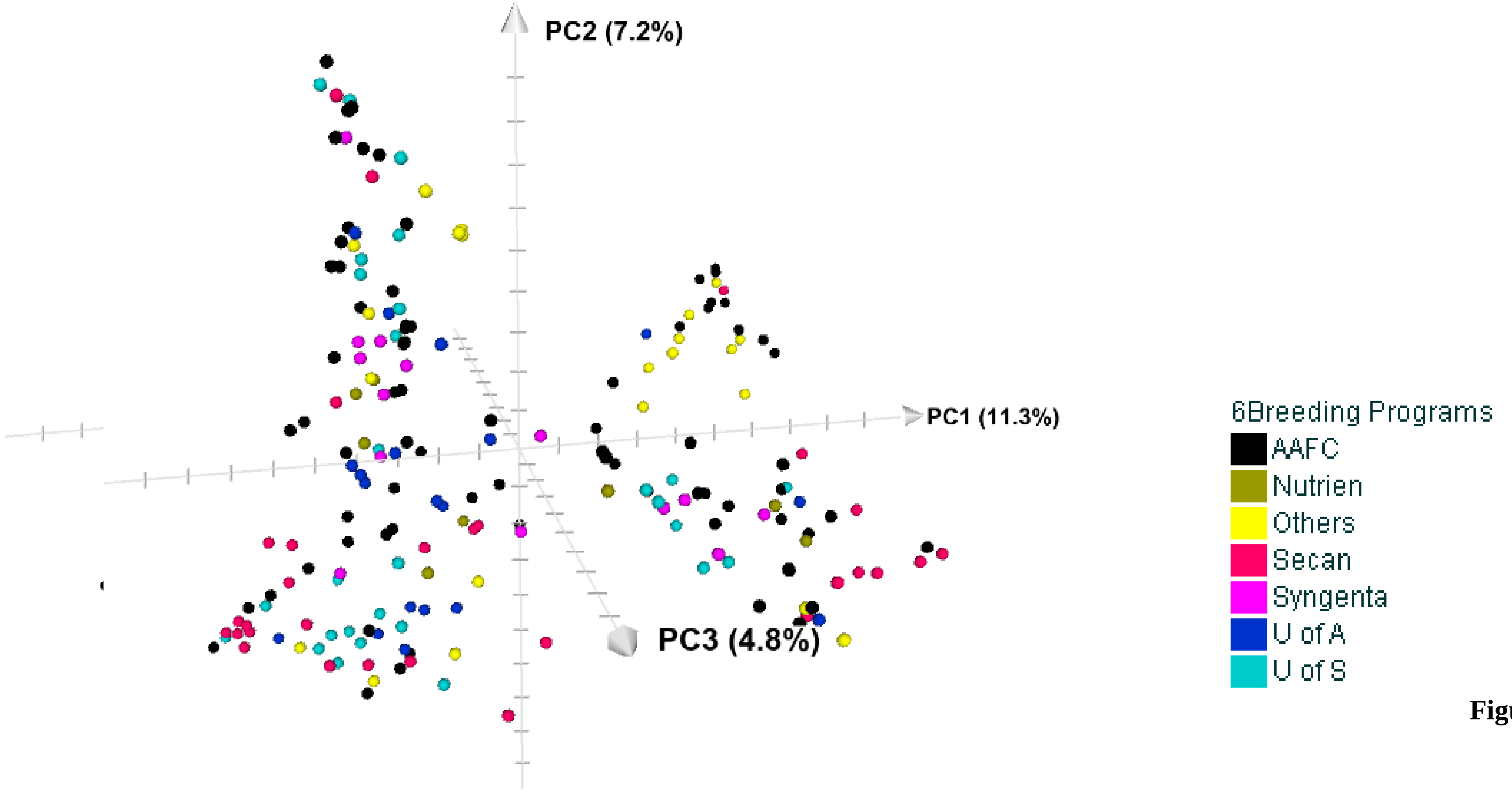

Figure S3 (continued)

Supplementary Figure S4. Neighbor-joining tree of 196 spring wheat cultivars genotypes based on identity by state based genetic distance matrix computed from 28,798 polymorphic SNPs. Groups were colored based on (a) eight wheat classes; (b) six breeding periods, (c) four breeding periods, and (d) six breeding programs (representative institutions).

(**a**) Eight wheat classes: CWRS (black), CNHR (blue), CPSR (red), CPSW (lime), CWES (olive), CWHWS (pink), CWSP (aqua), and CWSWS (maroon)

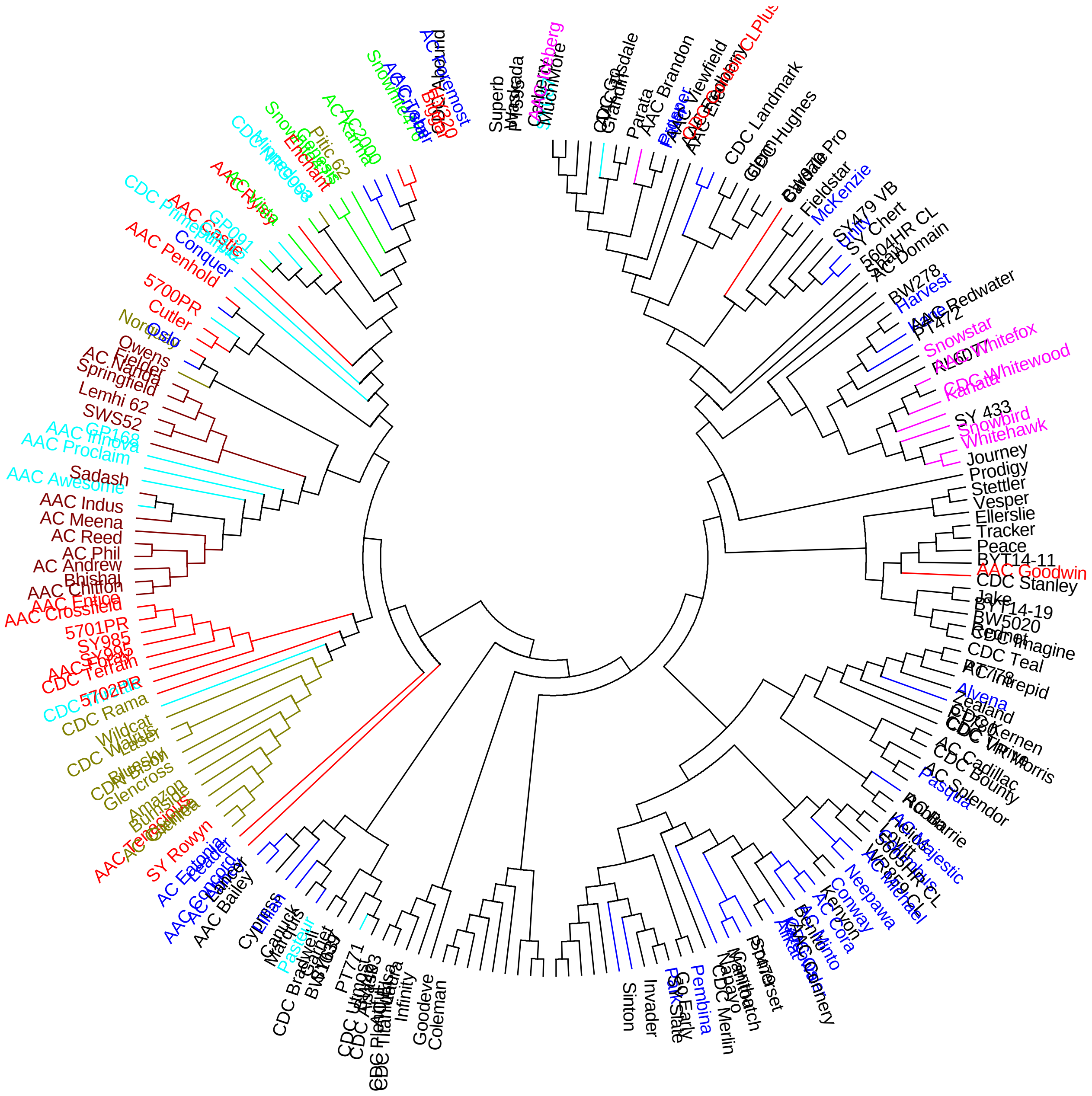

Figure S4

(**b**) Six breeding periods: 1905-1970 (blue), 1971-1980 (red), 1981-1990 (olive), 1991-2000 (green), 2001-2010 (purple), 2011-2018 (black), and (e) unknown (yellow).

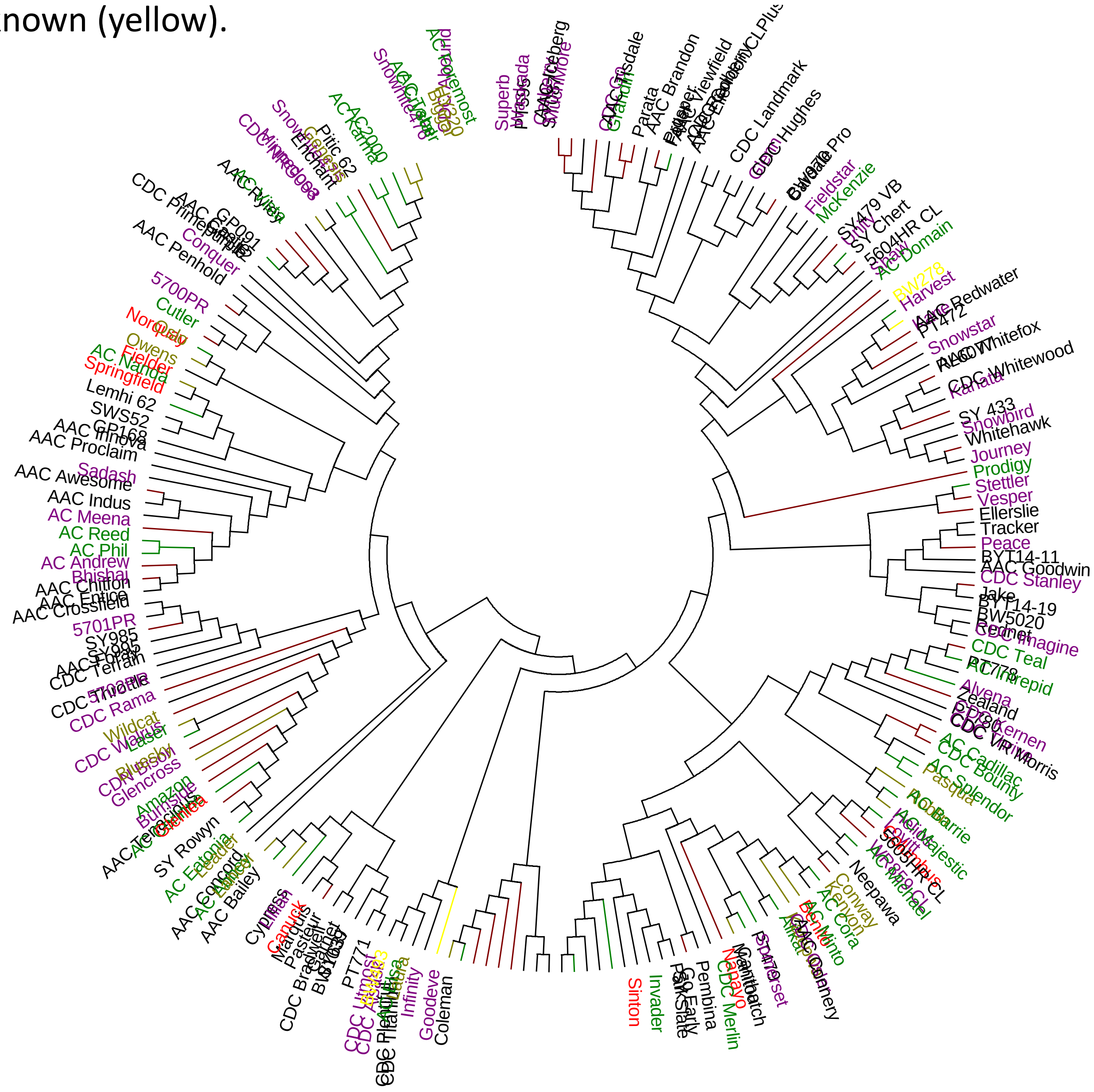

Figure S4 (continued)

(**c**) Four breeding periods: 1905-1970 (black), 1971-2000 (blue), 2001-2010 (red) and 2011-2018 (olive).

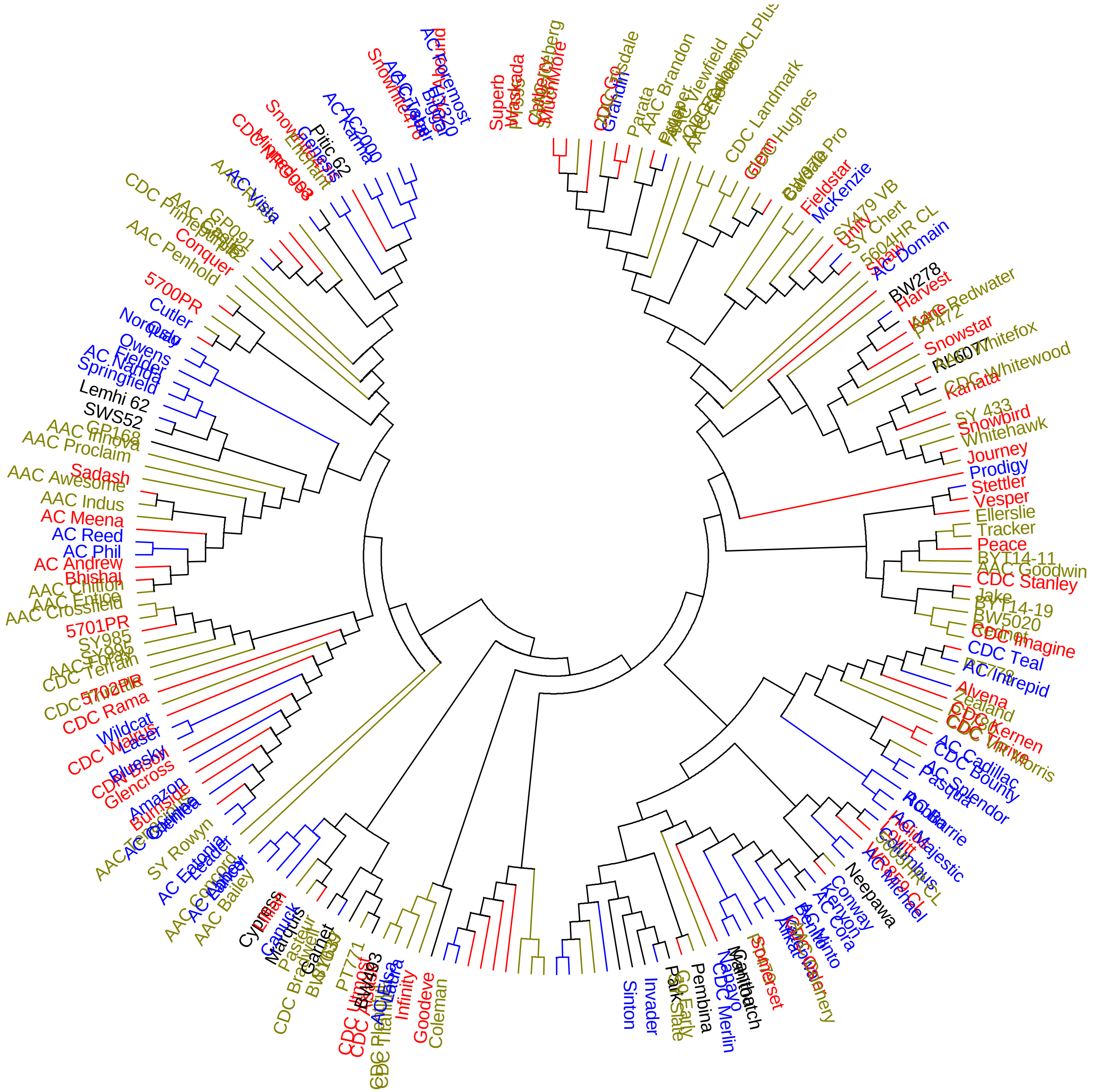

Figure S4 (continued)

(**d**) Six breeding programs (representative institutions): Agriculture and Agri-Food Canada (black), University of Saskatchewan (aqua), University of Alberta (blue), Secan Association (red), Syngenta Canada Inc. (pink), and Nutrien AG Solutions Inc. (olive), and others (yellow).

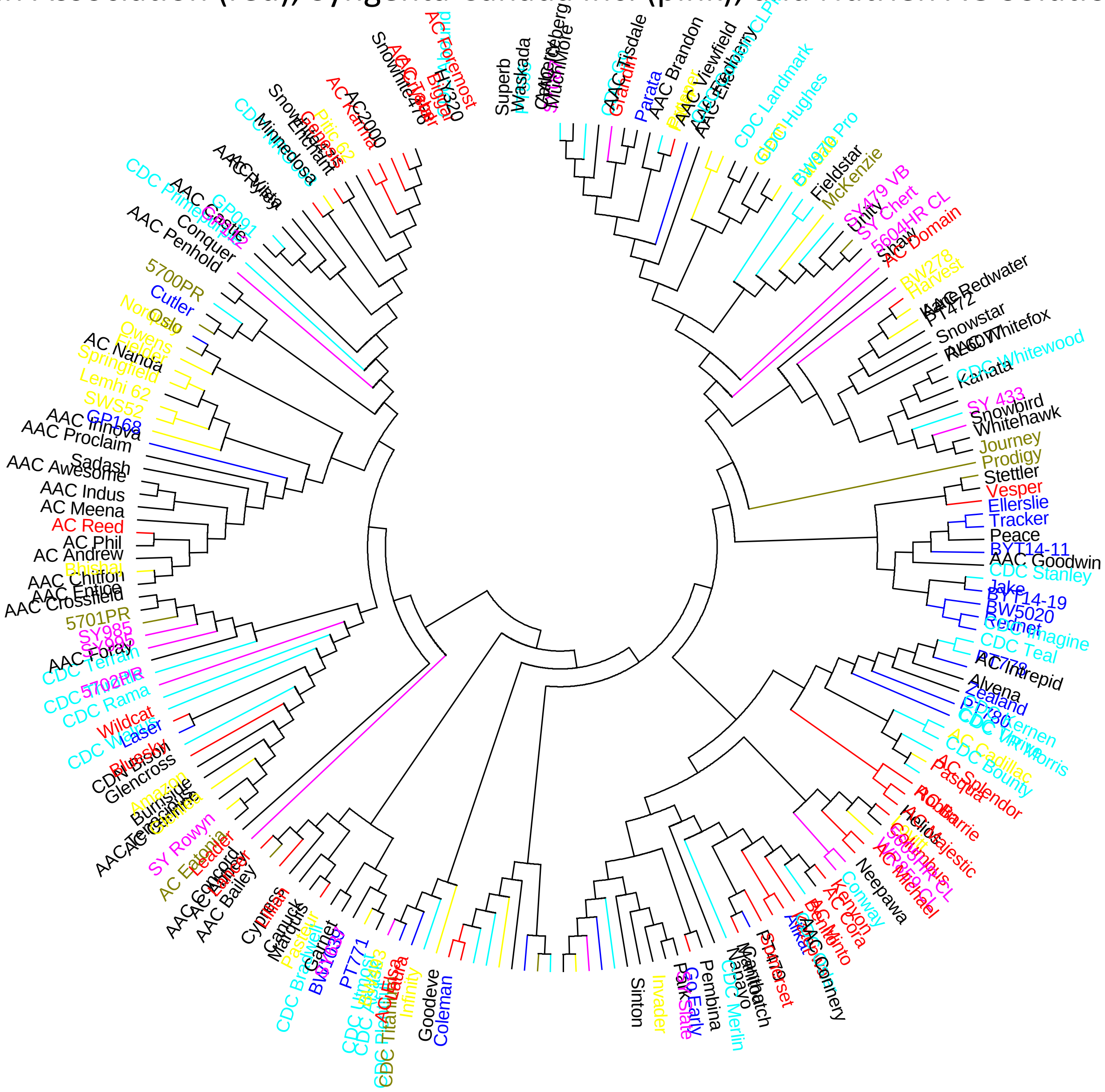

Figure S4 (continued)

Supplementary Figure S5. Genome-wide and chromosome-wise Manhattan plots of μ statistics from RAiSD against the physical map (Mb) based on the International Wheat Genome Sequencing Consortium (IWGSC) RefSeq v2.0. The horizontal line shows the threshold Mμ statistics for declaring selective sweeps on each chromosome.

Manhattan plot with the horizontal line showing the genome-wide threshold μ statistics at 26.0 for declaring candidate selective sweeps across all chromosomes. Using this genomewide threshold value, there are candidate selective sweeps only on chromosomes 2B, 3B, 4A, 4B, 7A, and 7B. ***However, we used chromosome-wide threshold values as shown in the next pages.*** See Supplementary Table S8 for details of each region.

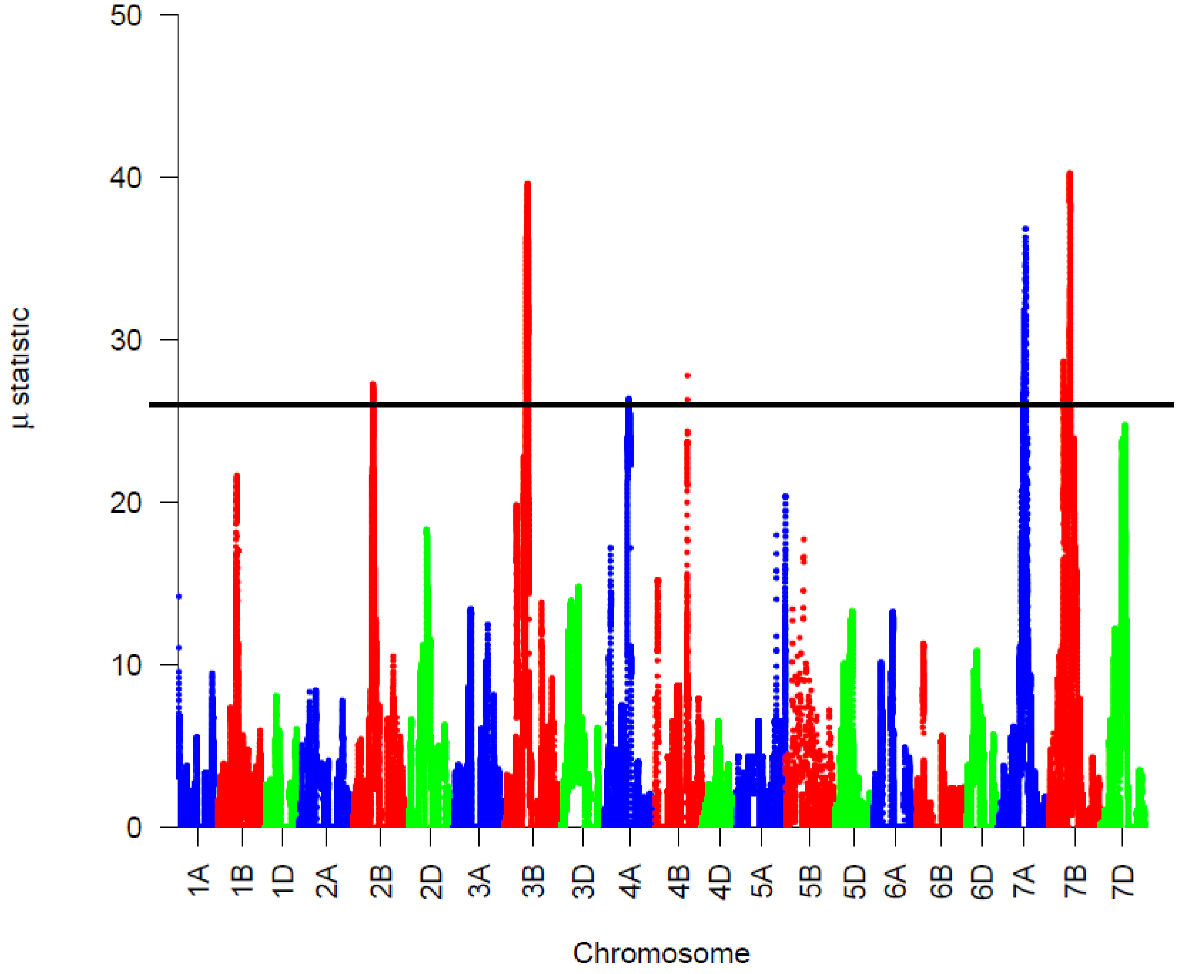

Figure S5

Chromosome-wise Manhattan plot

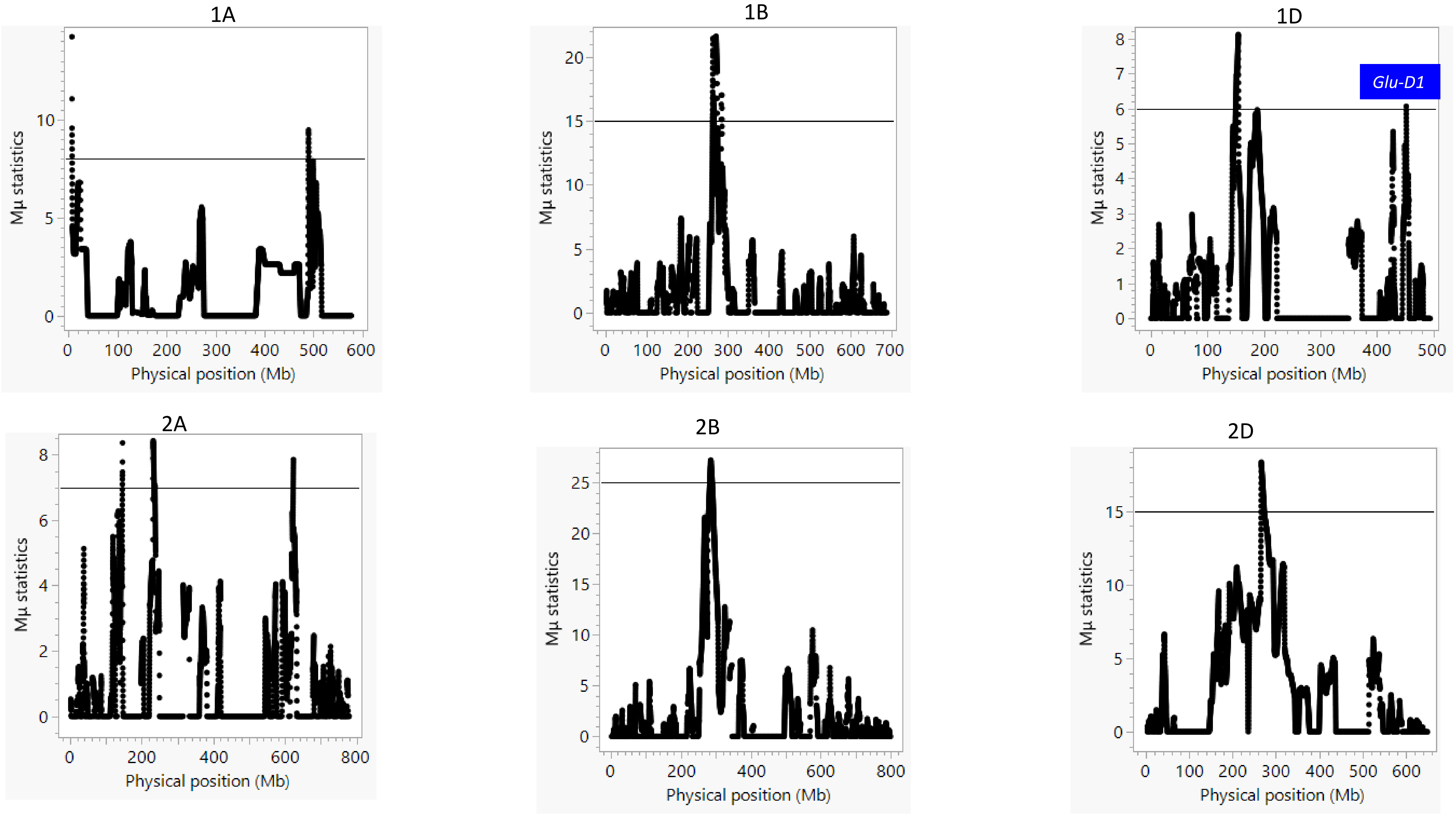

Figure S5 (continued

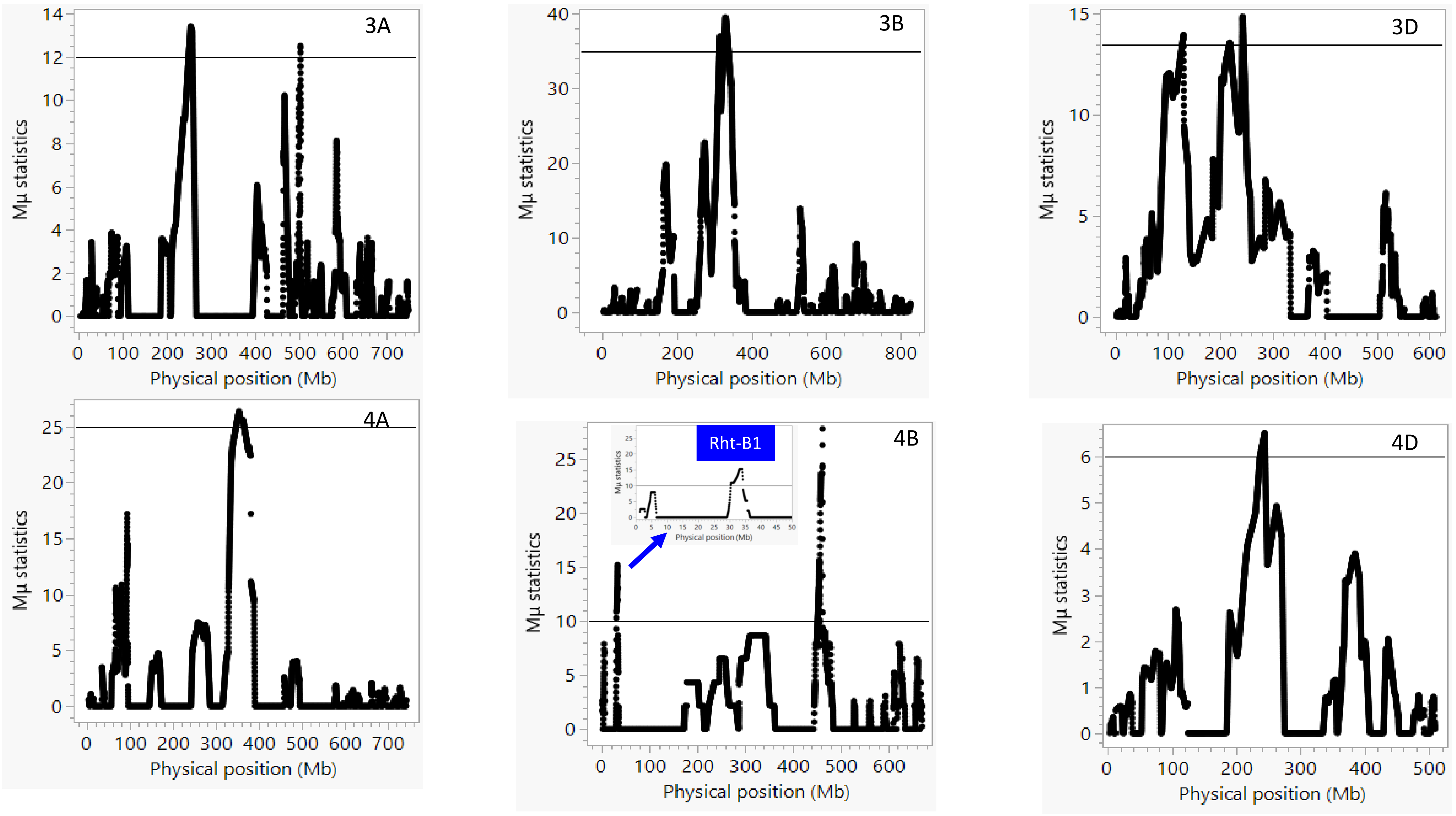

Figure S5 (continued)

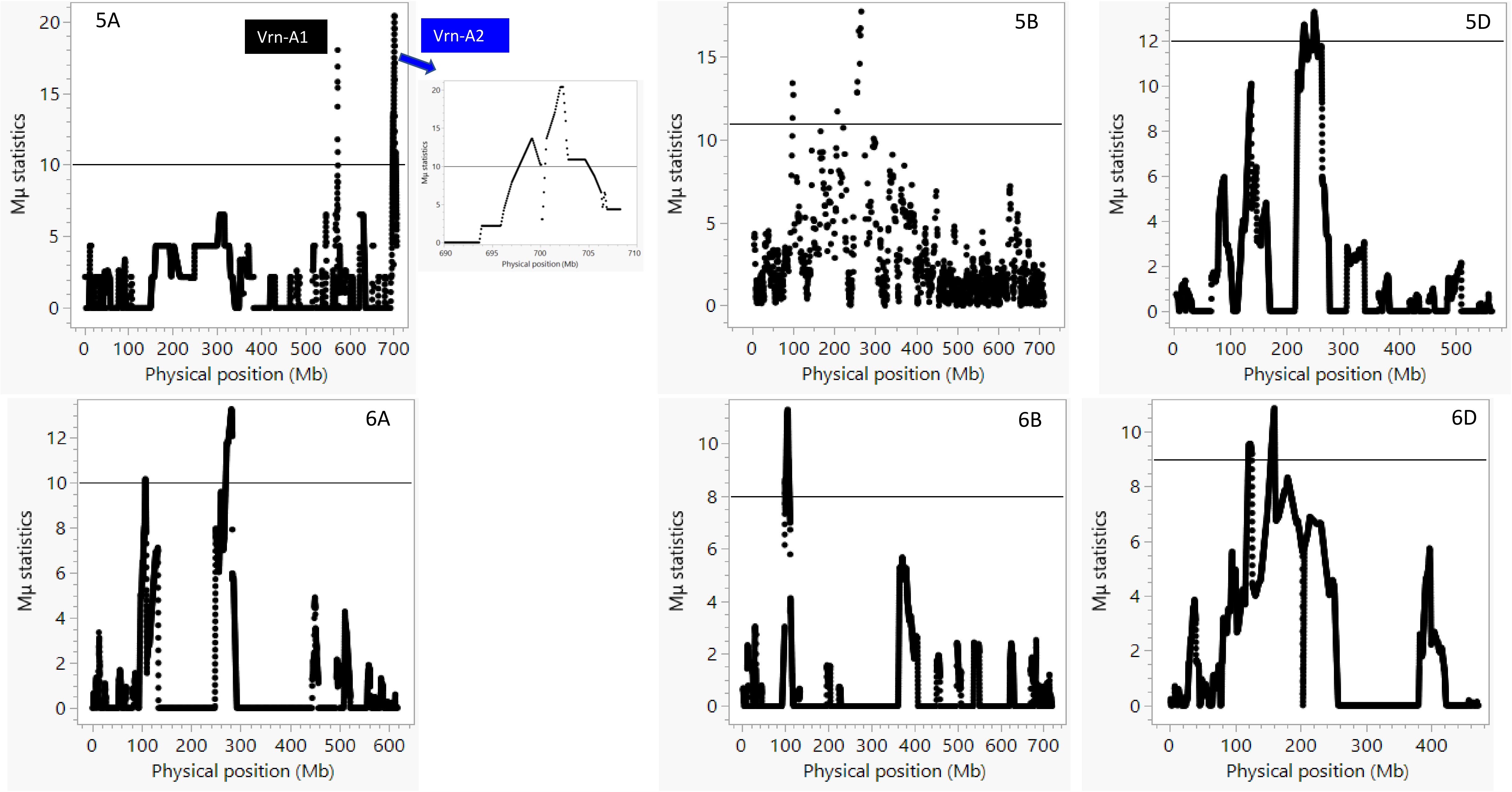

Figure S5 (continued)

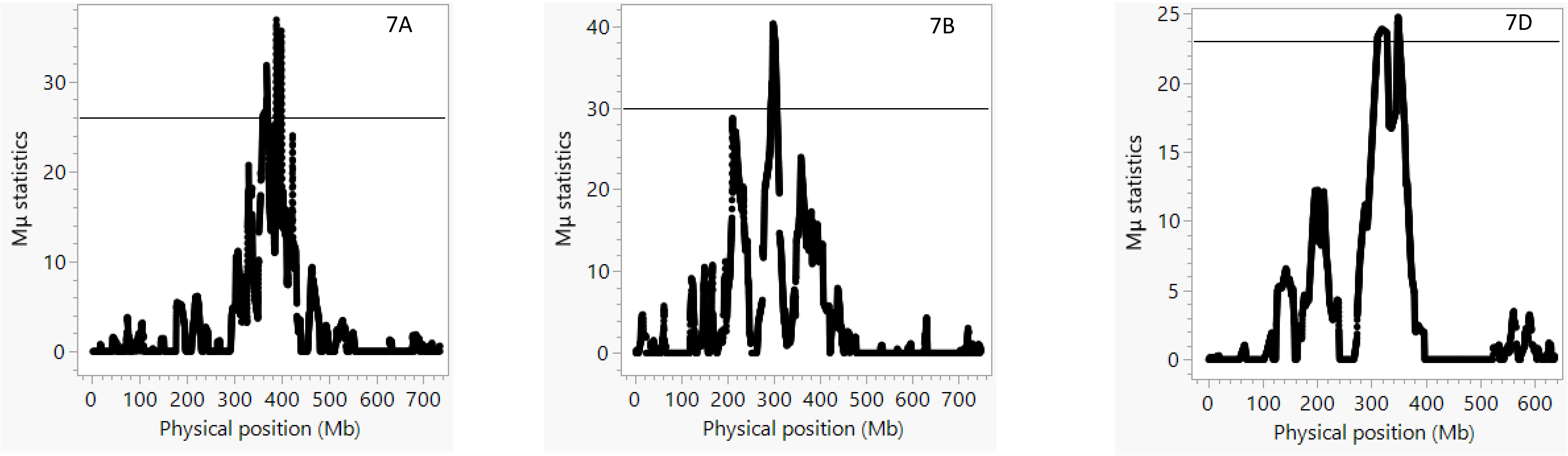

Figure S5 (continued)
